# Supplementary material for: The globalizability of temporal discounting
Source: Nat Hum Behav. 2022 Jul 11;6(10):1386–97. doi: 10.1038/s41562-022-01392-w (PMC9584811; doi:10.1038/s41562-022-01392-w)
Supplement: Supplementary file 1 — Supplementary Methods, Figs. 1–10 and Tables 1–18. [file 41562_2022_1392_MOESM1_ESM.pdf]

nature portfolio

https://doi.org/10.1038/s41562-022-01392-w

Supplementary information

The globalizability of temporal discounting

In the format provided by the authors and unedited

Supplementary Materials for

The Globalizability of Temporal Discounting

Kai Ruggeri, Amma Panin, Milica Vdovic, Bojana Većkalov, Nazeer Abdul-Salaam, Jascha Achterberg, Carla Akil, Jolly Amatya, Kanchan Amatya, Thomas Lind Andersen, Sibele D. Aquino, Arjoon Arunasalam, Sarah Ashcroft-Jones, Adrian Dahl Askelund, Nélida Ayacaxli, Aseman Bagheri Sheshdeh, Alexander Bailey, Paula Barea Arroyo, Genaro Basulto Mejía, Martina Benvenuti, Mari Louise Berge, Aliya Bermaganbet, Katherine Bibilouri, Ludvig Daae Bjørndal, Sabrina Black, Johanna K. Blomster Lyshol, Tymofii Brik, Eike Kofi Buabang, Matthias Burghart, Aslı Bursalıoğlu, Naos Mesfin Buzayu, Martin Čadek, Nathalia Melo de Carvalho, Ana-Maria Cazan, Melis Çetinçelik, Valentino E. Chai, Patricia Chen, Shiyi Chen, Georgia Clay, Simone D'Ambrogio, Kaja Damnjanović, Grace Duffy, Tatianna Dugue, Twinkle Dwarkanath, Esther Awazzi Envuladu, Nikola Erceg, Celia Esteban-Serna, Eman Farahat, Robert A., Farrokhnia, Mareyba Fawad, Muhammad Fedryansyah, David Feng, Silvia Filippi, Matías A. Fonollá, René Freichel, Lucia Freira, Maja Friedemann, Ziwei Gao, Suwen Ge, Sandra J. Geiger, Leya George, Iulia Grabovski, Aleksandra Gracheva, Anastasia Gracheva, Ali Hajian, Nida Hasan, Marlene Hecht, Xinyi Hong, Barbora Hubená, Alexander Gustav Fredriksen Ikonomeas, Sandra Ilić, David Izydorczyk, Lea Jakob, Margo Janssens, Hannes Jarke, Ondřej Kácha, Kalina Nikolova Kalinova, Forget Mingiri Kapingura, Ralitsa Karakasheva, David Oliver Kasdan, Emmanuel Kemel, Peggah Khorrami, Jakub M. Krawiec, Nato Lagidze, Aleksandra Lazarević, Aleksandra Lazić, Hyung Seo Lee, Žan Lep, Samuel Lins, Ingvild Sandø Lofthus, Lucía Macchia, Salomé Mamede, Metasebiya Ayele Mamo, Laura Maratkyzy, Silvana Mareva, Shivika Marwaha, Lucy McGill, Sharon McParland, Anișoara Melnic, Sebastian A. Meyer, Szymon Mizak, Amina Mohammed, Aizhan Mukhyshbayeva, Joaquin Navajas, Dragana Neshevska, Shehrbano Jamali Niazi, Ana Elsa Nieto Nieves, Franziska Nippold, Julia Oberschulte, Thiago Otto, Riinu Pae, Tsvetelina Panchelieva, Sun Young Park, Daria Stefania Pascu, Irena Pavlović, Marija B. Petrović, Dora Popović, Gerhard M. Prinz, Nikolay R. Rachev, Pika Ranc, Josip Razum, Christina Eun Rho, Leonore Riitsalu, Federica Rocca, R. Shayna Rosenbaum, James Rujimora, Binahayati Rusyidi, Charlotte Rutherford, Rand Said, Inés Sanguino, Ahmet Kerem Sarikaya, Nicolas Say, Jakob Schuck, Mary Shiels, Yarden Shir, Elisabeth D. C. Sievert, Irina Soboleva, Tina Solomonia, Siddhant Soni, Irem Soysal, Federica Stablum, Felicia T. A. Sundström, Xintong Tang, Felice Tavera, Jacqueline Taylor, Anna-Lena Tebbe, Katrine Krabbe Thommesen, Juliette Tobias-Webb, Anna Louise Todsen, Filippo Toscano, Tran Tran, Jason Trinh, Alice Turati, Kohei Ueda, Martina Vacondio, Volodymyr Vakhitov, Adrianna J. Valencia, Chiara Van Reyn, Tina A.G. Venema, Sanne Verra, Jáchym Vintr, Marek A. Vranka, Lisa Wagner, Xue Wu, Ke Ying Xing, Kailin Xu, Sonya Xu, Yuki Yamada, Aleksandra Yosifova, Zorana Zupan, Eduardo Garcia-Garzon

Correspondence to: kai.ruggeri@columbia.edu

Content

Supplementary Methods	Page 3
Supplementary Figures	Page 10
Supplementary Tables	Page 20

Methods

All materials and methods followed our pre-registered plan (osf.io/jfvh4). Significant deviations from the original plan are highlighted in each corresponding section, alongside the justification from the same.

Intertemporal decision measures involved mostly binary option sets between an immediate or closer-to-present option and a later or delayed option. Choice sets relied on base values using approximately 10% or 100% of the average (typically median) monthly household income for each country, using the local currency or whatever currency (often US$) is the standard in local social research and surveying. Language and general values were used based on recent studies that had a similar many-country approach and yielded reliable findings [1]. Those studies also influenced our decision to use values large enough that variability in the difference from 10% or 100% (or the alternate value) would be random between countries, constant within, and likely inconsequential to participants. They also convinced us to avoid values that appear to have some sort of implicit indications of 'good' or 'bad', hence keeping values typically in multiples of 10s, 100s, or 1000s, and timelines always built off of years rather than shifting between days, weeks, months, years, and beyond.

Definition of variables

Individual-level variables.

Age: Variable ranging from 18 to 100.

Education Completed. Variable presenting the highest level of education attained. This variable presented five groups: "Primary education or less", "Secondary education", "Technical or Vocational education"; "Bachelor studies or equivalent"; "Higher or Graduated degree".

Employment. Variable representing current employment status. Presented the following levels: "Employed full-time" (which included military service), "Employed part-time"; "Self-employed", "Not in paid employment but looking"; "Not in paid employment for personal reasons", "Full-time student" and "Retired".

Gender. Variable presenting three levels: "Male", "Female", and "Other". Other represented all individuals not categorizing themselves into strict male to female categories.

Individual income. Variable representing self-reported annual income from all sources before taxes.

Individual debt. Variable representing self-reported total debt balance as of June 30th, 2021 (including credit cards, student loans, and other credit not including housing or monthly bills, except if overdue).

Individual assets. Variable representing self-reported total assets including savings, retirement plans, investments accounts, and home equity as of June 30th, 2021.

Country-level variables

Gross domestic product (in current US$). Data obtained from World Bank database (https://data.worldbank.org/indicator/NY.GDP.MKTP.CD)

GINI index. World Bank estimate. We used the latest data available retrieved from https://data.worldbank.org/indicator/SI.POV.GINI

Inflation: We used inflation as relative in consumer prices index (change in annual percentage) from the World Bank database (retrieved from https://data.worldbank.org/indicator/FP.CPI.TOTL.ZG). If available, we employed the latest estimations obtained from official sources.

The survey included several additional variables (e.g., monthly discretionary spending) not covered in our analyses but included in the dataset and codebook for future analyses.

Data transformations

Following the pre-registration plan, we centered all variables according to their level of analysis (individual responses or country-level variables). We further standardize all variables (to present a mean of 0 and a standard deviation of 1) to simplify the interpretation of our results. All economic variables (e.g., assets or debt) were transformed from local currencies to dollars using exchange market rates collected on August 22nd, 2021, on Morningstar. We converted GDP to log GDP to facilitate the simpler interpretation of results, in line with standards in the field. Alternative transformations of other predictors were considered when plotting data and inspecting results (e.g., applying an inverse hyperbolic transformation to individual income inequality) but were discarded for our main analyses.

All main variables were computed as they were pre-registered, the only exception being the delay-speedup anomaly. Delay-speedup was originally intended to be measured using two scenarios, and however, we observed that the information provided by these items was redundant. In the end, we decided to calculate delay-speedup only using the simplified indifference point to the first gain block and the "speedup" item (asking participants to choose between receiving their indifference point in 12 months reducing an amount to receive only $500 now). This approach did not significantly impact the outcome but created a more reasonably sensitive measure for capturing the anomaly; the two similar items were notably more insensitive. We did not provide specification analyses as we observed that our main non-linear effects were invariant when specifying alternative controls.

Approach to scoring

We constructed a temporal discounting score that allows us to rank individuals by the number of immediate/earlier choices they made across all questions. We assigned individuals a score from zero to five for each question. Each question consisted of five sub-questions. Individuals saw at most three sub-questions depending on the order of their choices.

For example, in the US, participants first saw a choice between $500 immediately or $550 in one year. Participants who chose the immediate option were shown the same option set, but the delayed value was now $600. If they continued to prefer the immediate option, a final option offered $750 as the delayed reward. If participants choose the delayed option initially, subsequent choices were $510 and $505.

The constructed score corresponded to the ranking of the sub-question where the individual switched from choosing the delayed option towards the sooner option. An individual who always chose the later option was assigned a score of 0. An individual who always chose the sooner option was assigned a score of 5.

Adding this score across questions results in a total score of 0 for individuals always choosing the delayed amount and 19 for individuals always choosing the sooner amount. Therefore,

individuals with higher scores made more immediate choices. This scoring system allows us to compare discounting across all questions irrespective of the presence of other choice anomalies. The score also allows us to compare overall discounting across individuals as it simply encodes whether an individual made more or fewer immediate choices in the whole set of questions.

In this way, the score provides a useful means to summarize and compare the number of immediate choices made by individuals, an indicator of overall discounting behavior. It encodes this information for all questions and therefore does not depend on the presence or absence of specific choice anomalies. This suggests that it will be reliable in settings where the goal is to compare a summary measure of discounting across individuals, and where the score can be calculated from a series of choices, where the series is designed to capture a range of choice anomalies.

On a broad level, we consider the score itself to be a reliable measure (ω_{total}= .85). Given that most participants answered a set of different items, our estimation is based on the average block scores and responses to anomaly questions. In other words, our design precluded us to employ alternative measures of reliability, such as split-half reliability estimates, and, therefore, reliability estimates presented here should only be considered as an initial approximation.

Part of what makes this scoring approach valid is that exceptionally high scores will indicate individuals that are more likely to discount to extremes in all conditions, and vice versa for exceptionally low scores. Even if this is less clear for more moderate scores, hence including analysis of each anomaly for better precision, a more moderate score will at least indicate a normative overall choice/discount pattern. In other words, you may find similar scores for one person with slight discounting in all conditions and another person with extreme discounting in a few conditions but none in others. Across many choices, they would still arrive at similar outcomes, unlike those with extremely high and extremely low scores. In that way, the score is indicative of discounting broadly, irrespective of specific anomalies that may or may not have been observed for an individual.

There are multiple approaches to 'scoring' temporal discounting, and decomposing the concept broadly into subcomponents. In our case, we favored an atheoretical, simple approach to estimate effects. However, intertemporal discount rates have could also be explicitly modeled using novel developments that go beyond estimating discount rates employing an hyperbolic model [2,3]. Recent alternatives based on hierarchical models employing Bayesian estimation have been shown to be particularly informative [4,5] and should be considered for future exploration with ours or other data.

Specific country issues before data analyses

Georgia

We detected a coding issue for Georgia where incorrect values were shown in the first block of gain items. Participants who chose delayed gains should have seen the options "gain $500 now vs. $750 in 12 months". However, the later amount presented was incorrect. Thirty-four individuals were shown incorrect values for the third item of the first gain block (i.e., gain $500 now vs. $750 later). For those individuals, we imputed their responses by doing a weighted random sampling of responses from the remaining 152 individuals from Georgia. The weights ensured that the proportion of sooner and delayed responses remained unchanged before and after the imputation. We, therefore, stress some caution when using those data or given the large sample for Georgia, simply excluding those participants. These participants are indicated in the dataset.

Estonia

There was an error in Estonia's present bias and subadditivity anomalies items. Thus, those two anomalies were not computed for this country. As the responses to these two items are used to calculate the temporal discount scores, scores were calculated in Estonia's 0-17 scale and rescaled to 0-19 afterward. Estonia was accordingly removed in anomaly visuals related to these.

Japan

Japan was the only country were any participants (86.2%) received payment for participation. Based on local guidance, we used Yahoo! participant platforms. In comparing the paid and non-paid participants, we observed only small differences in average scores (Welch *t*-tests: t(86) = 2; p = 0.02), and no differences in present bias (proportion test with continuity correction: $\chi^2(1) = 2$; p = 0.24), absolute magnitude ($\chi^2(1) = 0.3$; p = 0.60), delay-speedup ($\chi^2(1) = 1*10^{-9}$; p = 0.99), gain-loss asymmetry ($\chi^2(1) = 1$; p = 0.3) or subadditivity ($\chi^2 (1) = 1*10^{-13}$; p = 1.00). Thus, we combined data from both paid and non-paid participants for our analyses, and include a variable to identify the data sources if future analysts are interested in studying further differences. These participants are indicated in the dataset.

Deviation from the pre-registered plan

We aimed to follow our pre-registration analyses as closely as possible. On certain selected occasions, we decided to amplify the scope of analyses and present robustness checks for the results presented by employing alternative estimation and inference techniques.

There was only one substantive deviation from our pre-registered analyses aside from the delay-speedup calculation. In the original plan, we planned to explore the role of financial status. In our final analysis, we employed individual assets and debts to this end. Assets and debts were included as raw indicators instead of inequality measures as we did not find reliable national average assets or individual debt sources.

One minor adaptation from our pre-registration involved our plan to test for non-linear effects and use Bayesian estimation only as part of our exploratory analyses. However, as we identified several relevant non-linear effects, we modified our workflow to accommodate those as follows: a) we initially explored non-linear effects using hierarchical generative additive (mixed) models (GAMs); b) we included relevant non-linear effects in our main pre-registered models; c) we estimated Bayesian versions of these same models to test for whether null effects could be supported in certain cases.

Extended results

These extended results contain additional text to complement the main analyses and information on robustness checks not provided in the main text.

Further details on modeling temporal discounting

We found that a simpler model only fitting random intercept was adequate for scores and anomalies (Supplementary Table 5). As expected, models including random effects to estimate maximal models or reduced models including random effects for or main variables (inequality, GINI, assets, and debt) produced singular fit and convergence issues when using fixed linear models. Given the correspondences between GAM and mixed linear models, we used the former to study the fit of several models (null model, single random intercept model, main predictors as random intercept, and a full model including main predictors also as random slopes, Supplementary Table 5). χ^2 fREML scores test (corrected for effective degrees of freedom) favoring the random slopes model only for present bias, delay-speedup, and subadditivity. However, in all these cases, AIC indicated the simpler model should be retained. We observed no significant random slope smooth effect (at $p = 0.01$ level) in either of these anomalies, thus favoring the simpler models. In the end, we decided to retain the simpler random intercept model as expected in our pre-registered analyses,

All models show good convergence and no major deviations from assumptions. To interpret the results inspected the expected degrees of freedom (edf) and a corresponding p-value from the Wald test. The edf can be understood as an approximation to the smooth curvature, where zero values indicate a lack of association, values close to one indicate that the relationship is linear. Higher values indicate that the relationship between the variables becomes increasingly non-linear [41]. However, to better understand our results, we resorted to estimated conditional effects (Supplementary Figures 1-2).

Results from our initial GAM models Supplementary Table 6) revealed that, for temporal discount scores, three effects were highly non-linear: debt (edf =4.97, $p < 0.0001$), assets (edf = 4.96, $p < 0.0001$), and inflation (edf = 2.55, $p < 0.0001$). GINI was observed to be closer to be a linear effect (edf = 1.69, $p = 0.0028$), and it was modeled as such. Thus, we only included debt, assets, and inflation as smooths terms in our final mixed linear modes. For anomalies, we observed that assets and inflation were consistently identified as relevant non-linear effects in all cases but for assets in gain-loss asymmetry (edf = 0.78, $p = 0.041$) and subadditivity (edf = 0.001, $p = 0.418$). In the end, we decided to included debt and assets as smooth terms in our final models for all anomalies for consistency's sake. Individual income inequality presented two significant effects on present bias (edf = 0.95, $p = 0.009$) and absolute magnitude (edf = 1.03, $p = 0.004$). As these terms seemed to be highly linear (edf close to 1), we included this effect as such. Nevertheless, our main results remained invariant if alternative splines were selected (e.g., thin-plate splines, Supplementary Table 7). We evaluated model convergence and model fit via residual inspection.

A noteworthy effect might be the explained variance, which could be considered as low for the anomalies. When observing individual model predictions in analogous studies (see Falk & Hermle, 19, Supplementary Table 13), we found similar rates of explained variance.

All linear and generalized mixed models converged adequately (in both their non-Bayesian and Bayesian estimates). Some Bayesian models (for temporal scores, absolute magnitude, delay-speedup, and subadditivity) indicated that some transitions were divergent after warmup. However, as the number of divergent transitions never was higher than 10 (for temporal discount scores), and $\hat{R}$ hat, bulk, and tail effective sample size indicated correct convergence and correct parameter estimation, we decided to interpret those models. Moreover, we observed that our results were similar in our frequentist and

Bayesian estimated versions (Supplementary Table 8-Supplementary Table 11). To facilitate the interpretation of the results, we illustrated the smooth effects of GINI, assets, and debt on temporal discount scores (Supplementary Figure 1) and for assets and debt on the anomalies (Supplementary Figure 2).

Bayesian analysis of null effects of inequality

In our main analyses, we highlighted that individual income inequality neither predicted temporal discount scores nor anomalies except for present bias (OR = 1.07, 95% CI [1.03 – 1.13], p = 0.0006) and absolute magnitude (OR = 0.92, 95% CI [0.87 – 0.98], p = 0.0006). Similarly, we found a small effect of GINI on temporal discount scores (β = 0.09, 95% CI [0.02 – 0.16], p = 0.002), but not in the case of anomalies. Taking advantage of our Bayesian estimation effects, we presented a more detailed understanding of these effects. Firstly, it's noteworthy that results from the Bayesian models were similar, and in both cases, 95% credible intervals did not contain the point null effect (1 and 0, respectively). However, we decided to employ log Bayes factors and the percentage of posterior samples within a range of null effects to understand the support for a range null effect (Supplementary Table 12).

For the effect of GINI on temporal discount scores, we observed that half of the posterior samples (56%) were inside of a |0.10| range. This evidence indicates that in half of our posterior distribution estimation, the median effect of GINI was smaller than ±.10. Given the strong support for the null effect (BF_{01} = 48), this effect could be considered weak. A similar conclusion was reached for the effect of GINI on the present bias (63% of the posterior samples showed effects were inside the range |0.10|, BF_{01} = 59) and for gain-loss asymmetry (68% of the posterior samples indicated effects inside the range |0.10|, BF_{01} = 77).

For the effects of individual inequality on present bias and absolute magnitude, we found that around 86% and 71% of the posterior samples indicating null effects (BF_{01} = 250 and BF_{01} = 91, respectively). Thus, it is probable that the effect of individual economic inequality on the present bias will be largely inconsequential.

Robustness checks

Smooth effects ultimately depend upon choosing an appropriate number of knots to estimate the basic function of the non-linear effect [40-43]. Choosing an appropriate number of knots is thus necessary for the model to represent the underlying function adequately. Our analyses selected the number of knots in our final models based on the knots automatically identified by our original GAM estimation (using regularized penalized cubic splines). We further explored whether our results were sensitive to the selection of knots by increasing the number of knots defined in our final models. We did not focus on the exact estimated parameters (as for smooth splines, they are seldom interpretable), but rather on the estimated smooth functional form and conditional effects estimated made by each model. Conditional plots for smooth effects (Supplementary Figure 10) revealed no major differences when we increased the knots to 9, indicating that our method to select the smooth dimensionality was effective. Results for anomalies were similar and were left unreported.

Detailed results for Figures 2-5

Figure 2. included the conditional effect of standardized GINI on individual temporal discount scores and the normalized number of engaged anomalies per person. Estimated effects were obtained as conditional effects from the corresponding GAM models and presented in Supplementary Tables 10 and 13. Overall, and as observed in our primary analyses, we observed a negative, linear relationship between GDP temporal discount scores (β= 0.09, 95% CI [0.02, 0.06], p = 0.002). In the case of anomalies, we found no effect (β= 0.001, 95% CI [-0.03, 0.03], p = 0.936).

In Figure 3. we explored whether GDP was related to each of its main components (blocks of items regarding gains, payments, and large gains, scored 0 to 5) separately. Firstly, it should be noted that scores were higher for gain items than payments in the general sample and larger gains. Still, there existed strong variability between countries in this regard (Supplementary Table 4). Secondly, we tested whether (log) GDP could explain these differences by estimating a GAM model including GDP as a predictor of the scores of each block (Supplementary Table 14). We observed that GDP was strongly, non-linearly related with larger gains ($p < 0.001$), and to an extent, with gains ($p = 0.0041$). However, the relationship between GDP and payments was not significant ($p = 0.248$). Thus, country GDP is strongly related to gains (where higher GDP countries tend to present lower scores) than to payments.

In Figure 4. we provide descriptive information for temporal discount scores and anomalies. Additional, descriptive information detailed information on the random effects meta-analysis results in Supplementary Figures 3-8, including by-country results and prediction intervals. In addition, we were interested in knowing how many anomalies participants tend to show. Fifty-four per cent of participants showed at least one anomaly, with 33% presenting multiple, yet only 2% showing four (Supplementary Table 14). However, we also observe substantial variability between countries for these rates.

Lastly, in Figure 5, we explored the effect of wealth, debt inequality on temporal discount scores at country-level. To this end, we estimated six GAM models (with similar specifications to our principal models), including each of our main predictors separately (Supplementary Table 15). Again, most of the relationships found at the individual level translated when aggregating the results by country. We observed strong non-linear effects for inflation (edf = 2.43; $p < 0.001$) and assets (edf =1.91; $p = 0.0002$). We also observed a mild effect of GINI (edf = 1.1456; $p = 0.0054$) and GDP (edf = 1.027; $p = 0.0089$). We decided not to average debt at the country level but rather to study debt to national net average income ratio. In this case, and in contrary to what happened with individual debt levels, we observed that the debt to average income ratio did not predict scores (edf = 0.001; $p = 0.589$).

Given these results, we decided to employ the World Bank per-country classification in all the presented graphics to illustrate the potential effects of country economic conditions in our main results. As we plan to control GDP and individual income (via individual income inequality) in our analyses, we decided to follow our pre-registration and not include this new variable information in our main models.

Supplementary Figure 1. Effect of inflation, debt, and assets on temporal discount scores.

Conditional smooth effect of inflation (A), debt (B), and assets (C) on temporal discount scores. Results were estimated controlling for age, education, employment, GDP, and GINI. Temporal discount scores are standardized. 95% confidence intervals are printed in blue. Positive effect indicates an increasing temporal discounting, and negative effects indicate lower temporal discount effect.

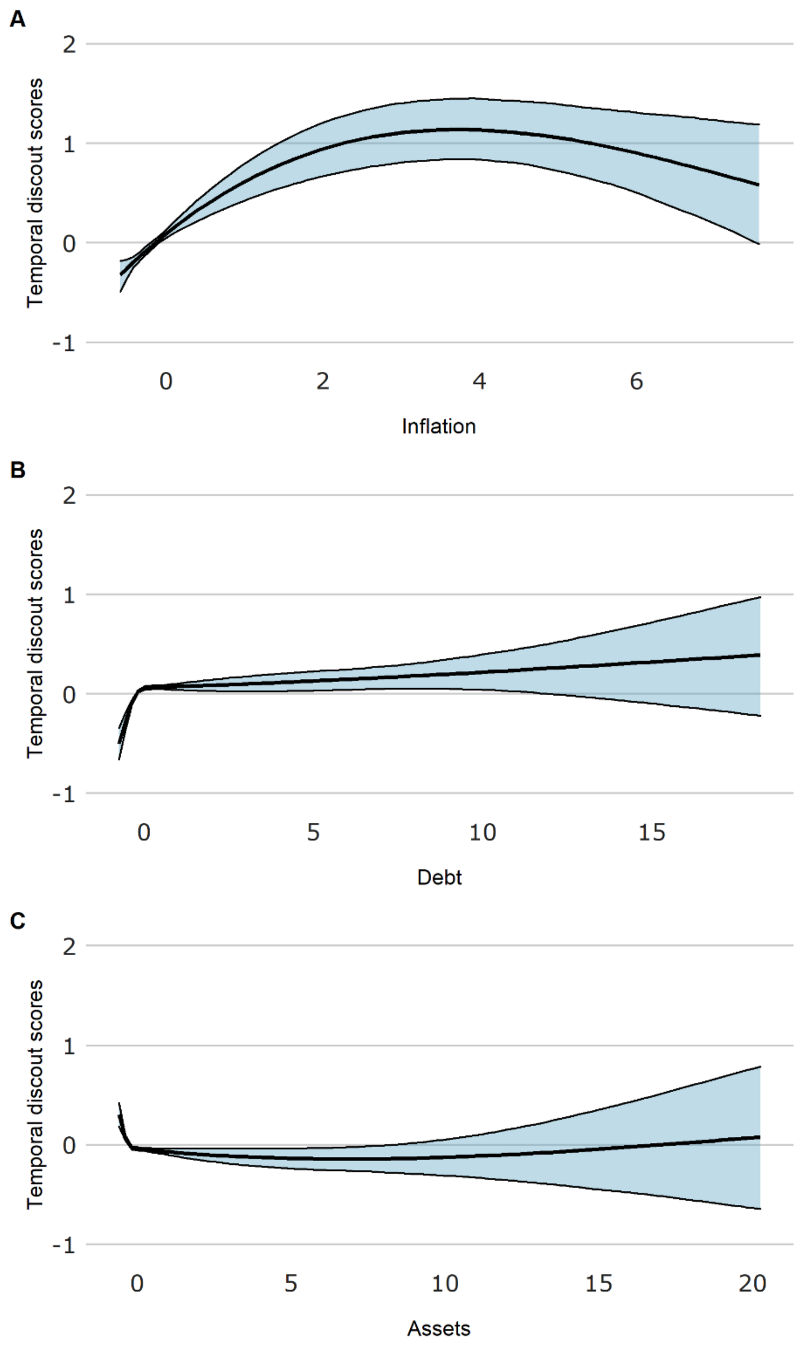

Supplementary Figure 2. Country-level effects of country-inequality on scores, scores per type of item, and anomalies per country.

Conditional smooth effect of inflation (A), debt (B), and assets (C) on temporal discount scores. Results were estimated controlling for age, education, employment, GDP, and GINI. Temporal discount scores are standardized, with inflation and assets being mean-centered per country and scaled. 95% confidence intervals are printed in blue. A positive effect indicates a higher likelihood of engaging on the anomaly, and negative effects indicate a lower likelihood of engaging.

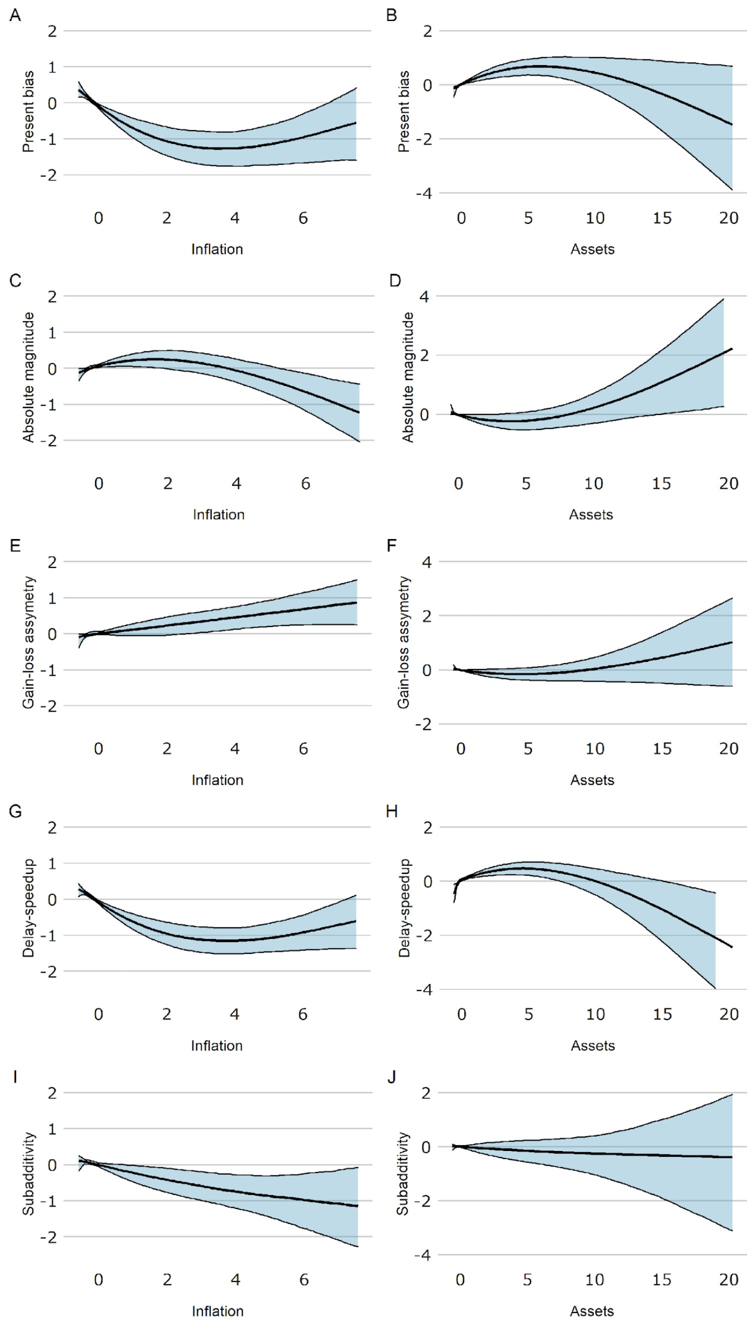

Supplementary Figure 3. Meta-analysis results for temporal discount scores.
Random effect meta-analysis on the country average temporal discount score. MRAW: sample mean. The overall random effect, prediction interval, and the I^2 heterogeneity test are presented at the bottom of the figure. The prediction interval is presented in red. Higher means indicate a higher temporal discount.

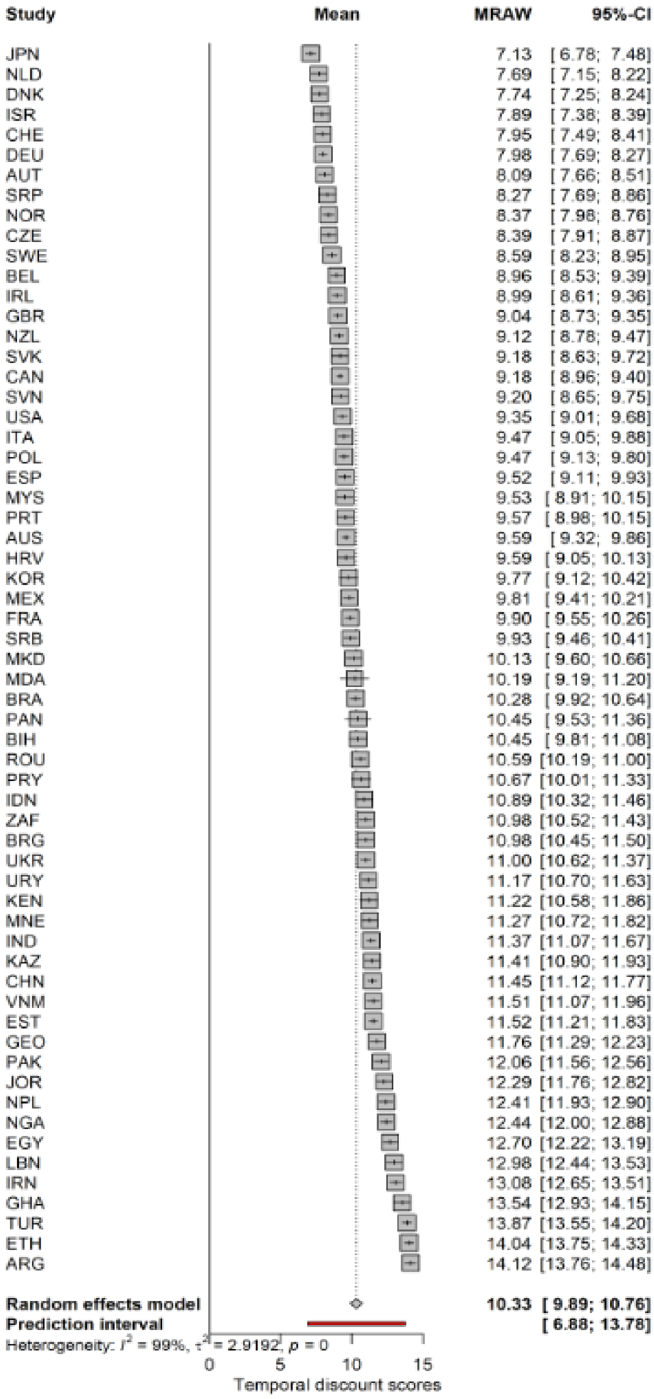

Supplementary Figure 4. Meta-analysis results for present bias.

Random effect meta-analysis on the country average temporal discount score. MRAW: sample mean. The overall random effect, prediction interval, and the I^2 Heterogeneity test are presented at the bottom of the figure. The prediction interval is presented in red. A higher proportion indicates a higher rate of engaging in the anomaly.

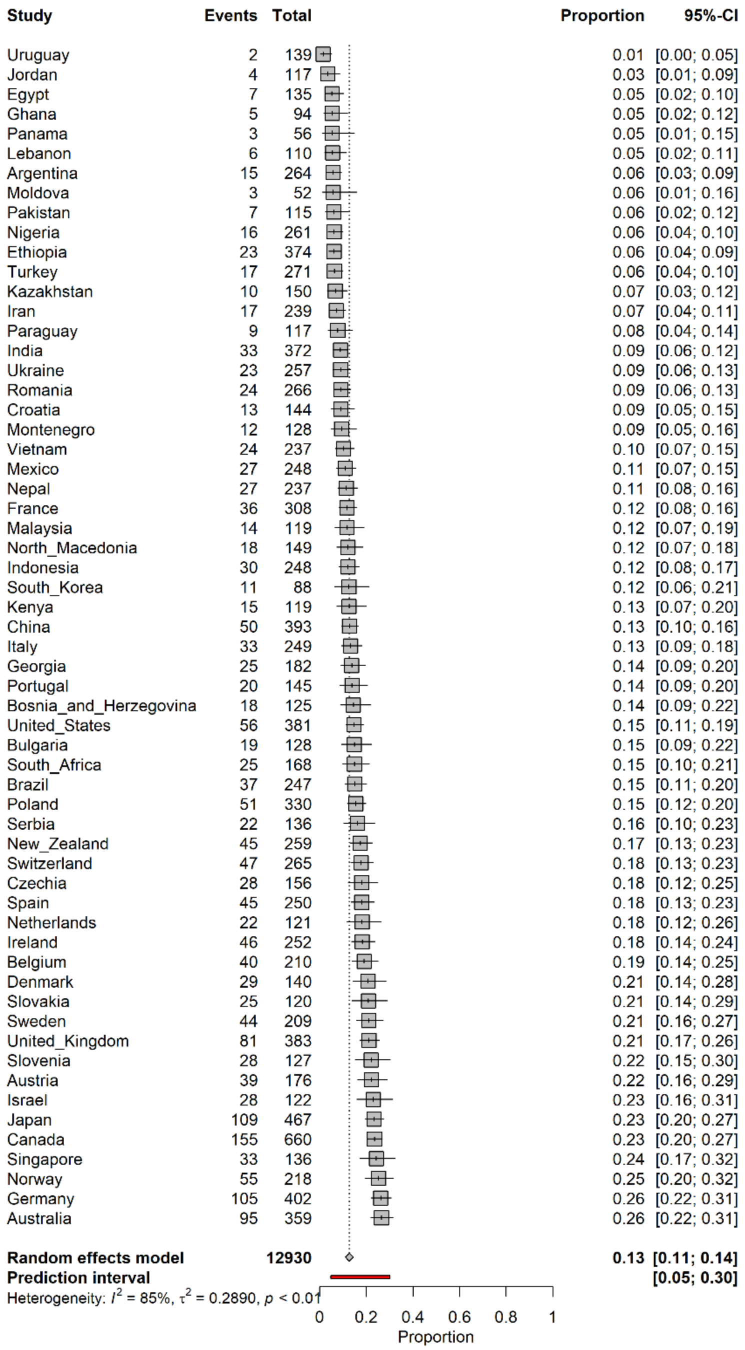

Supplementary Figure 5. Meta-analysis results for absolute magnitude

Random effect meta-analysis on the country average temporal discount score. MRAW: sample mean. The overall random effect, prediction interval, and the I^2 Heterogeneity test are presented at the bottom of the figure. The prediction interval is presented in red. A higher proportion indicates a higher rate of engaging in the anomaly.

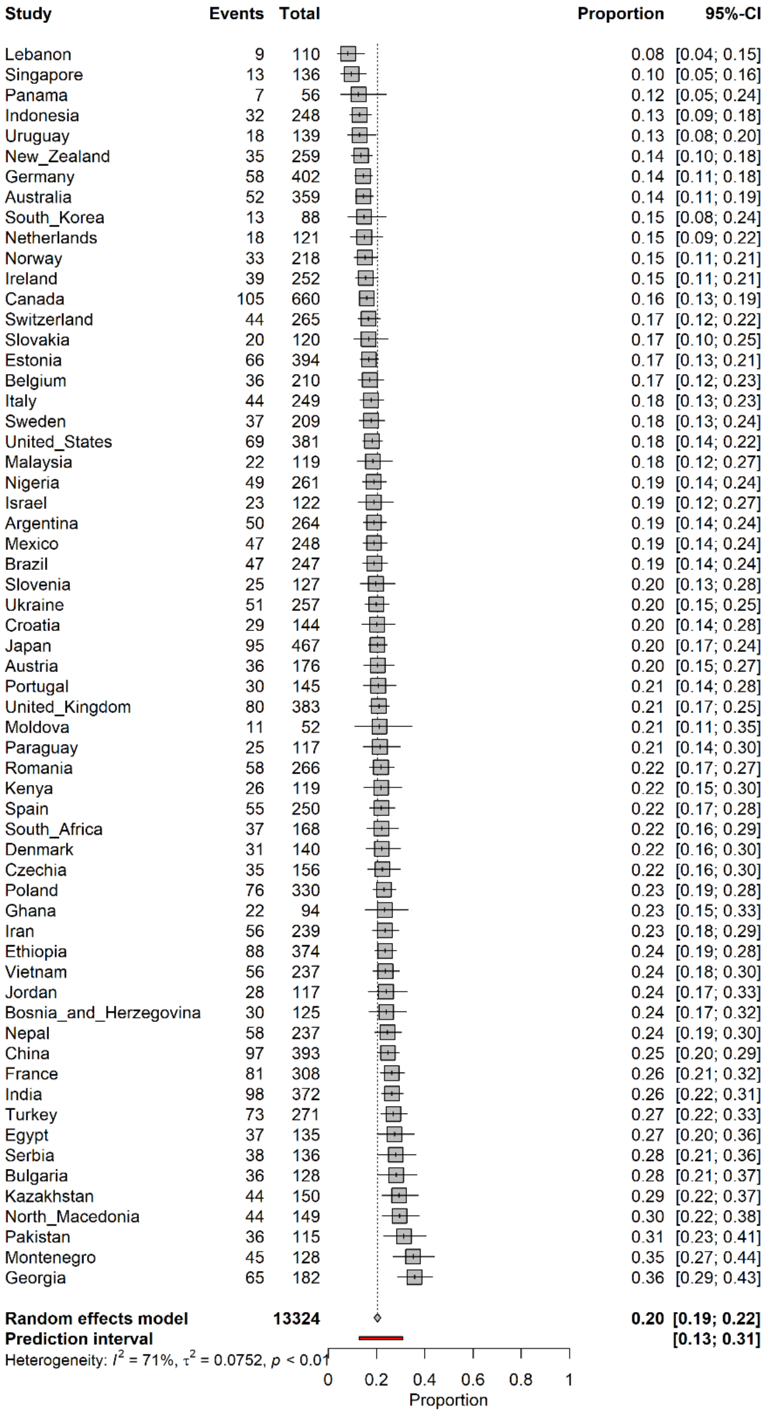

Supplementary Figure 6. Meta-analysis results for gain-loss asymmetry.

Random effect meta-analysis on the country average temporal discount score. MRAW: sample mean. The overall random effect, prediction interval, and the I^2 Heterogeneity test are presented at the bottom of the figure. The prediction interval is presented in red. A higher proportion indicates a higher rate of engaging in the anomaly.

Study	Events	Total	Proportion	95%-CI
Uruguay	35	139	0.25	[0.18; 0.33]
Panama	15	56	0.27	[0.16; 0.40]
Singapore	37	136	0.27	[0.20; 0.35]
South_Africa	46	168	0.27	[0.21; 0.35]
Australia	104	359	0.29	[0.24; 0.34]
Canada	194	660	0.29	[0.26; 0.33]
South_Korea	26	88	0.30	[0.20; 0.40]
New_Zealand	77	259	0.30	[0.24; 0.36]
Italy	78	249	0.31	[0.26; 0.37]
Belgium	67	210	0.32	[0.26; 0.39]
Paraguay	38	117	0.32	[0.24; 0.42]
Mexico	81	248	0.33	[0.27; 0.39]
Brazil	81	247	0.33	[0.27; 0.39]
Ireland	84	252	0.33	[0.28; 0.40]
Argentina	91	264	0.34	[0.29; 0.41]
Estonia	136	394	0.35	[0.30; 0.39]
Switzerland	92	265	0.35	[0.29; 0.41]
United_States	133	381	0.35	[0.30; 0.40]
Israel	43	122	0.35	[0.27; 0.44]
Spain	89	250	0.36	[0.30; 0.42]
Czechia	56	156	0.36	[0.28; 0.44]
Ethiopia	138	374	0.37	[0.32; 0.42]
Ukraine	95	257	0.37	[0.31; 0.43]
Slovenia	47	127	0.37	[0.29; 0.46]
Norway	82	218	0.38	[0.31; 0.44]
France	116	308	0.38	[0.32; 0.43]
Malaysia	45	119	0.38	[0.29; 0.47]
Portugal	55	145	0.38	[0.30; 0.46]
China	152	393	0.39	[0.34; 0.44]
United_Kingdom	149	383	0.39	[0.34; 0.44]
Germany	160	402	0.40	[0.35; 0.45]
Japan	188	467	0.40	[0.36; 0.45]
North_Macedonia	61	149	0.41	[0.33; 0.49]
Poland	136	330	0.41	[0.36; 0.47]
Vietnam	98	237	0.41	[0.35; 0.48]
Sweden	88	209	0.42	[0.35; 0.49]
Egypt	57	135	0.42	[0.34; 0.51]
Iran	101	239	0.42	[0.36; 0.49]
Austria	75	176	0.43	[0.35; 0.50]
Nepal	102	237	0.43	[0.37; 0.50]
Croatia	62	144	0.43	[0.35; 0.52]
Montenegro	56	128	0.44	[0.35; 0.53]
Netherlands	53	121	0.44	[0.35; 0.53]
Turkey	119	271	0.44	[0.38; 0.50]
Slovakia	53	120	0.44	[0.35; 0.54]
Bulgaria	57	128	0.45	[0.36; 0.54]
Serbia	61	136	0.45	[0.36; 0.54]
Romania	121	266	0.45	[0.39; 0.52]
Moldova	24	52	0.46	[0.32; 0.61]
Jordan	55	117	0.47	[0.38; 0.56]
Denmark	66	140	0.47	[0.39; 0.56]
Pakistan	55	115	0.48	[0.38; 0.57]
Bosnia_and_Herzegovina	60	125	0.48	[0.39; 0.57]
Kazakhstan	73	150	0.49	[0.40; 0.57]
Ghana	46	94	0.49	[0.38; 0.59]
Kenya	59	119	0.50	[0.40; 0.59]
India	192	372	0.52	[0.46; 0.57]
Indonesia	131	248	0.53	[0.46; 0.59]
Nigeria	139	261	0.53	[0.47; 0.59]
Georgia	114	182	0.63	[0.55; 0.70]
Lebanon	74	110	0.67	[0.58; 0.76]
**Random effects model**		**13324**	**0.40**	**[0.38; 0.42]**
**Prediction interval**				**[0.26; 0.55]**

Heterogeneity: $I^2 = 81\%$, $\tau^2 = 0.0910$, $p < 0.01$

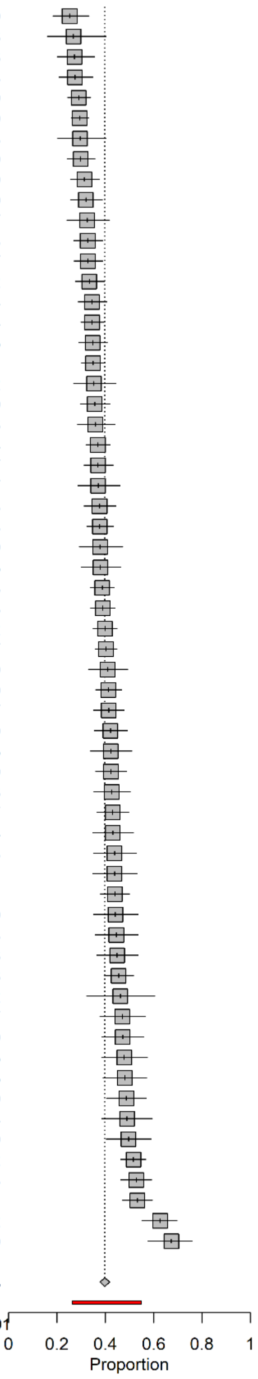

Supplementary Figure 7. Meta-analysis results for delay-speedup anomaly.

Random effect meta-analysis on the country average temporal discount score. MRAW: sample mean. The overall random effect, prediction interval, and the I^2 Heterogeneity test are presented at the bottom of the figure. The prediction interval is presented in red. A higher proportion indicates a higher rate of engaging in the anomaly.

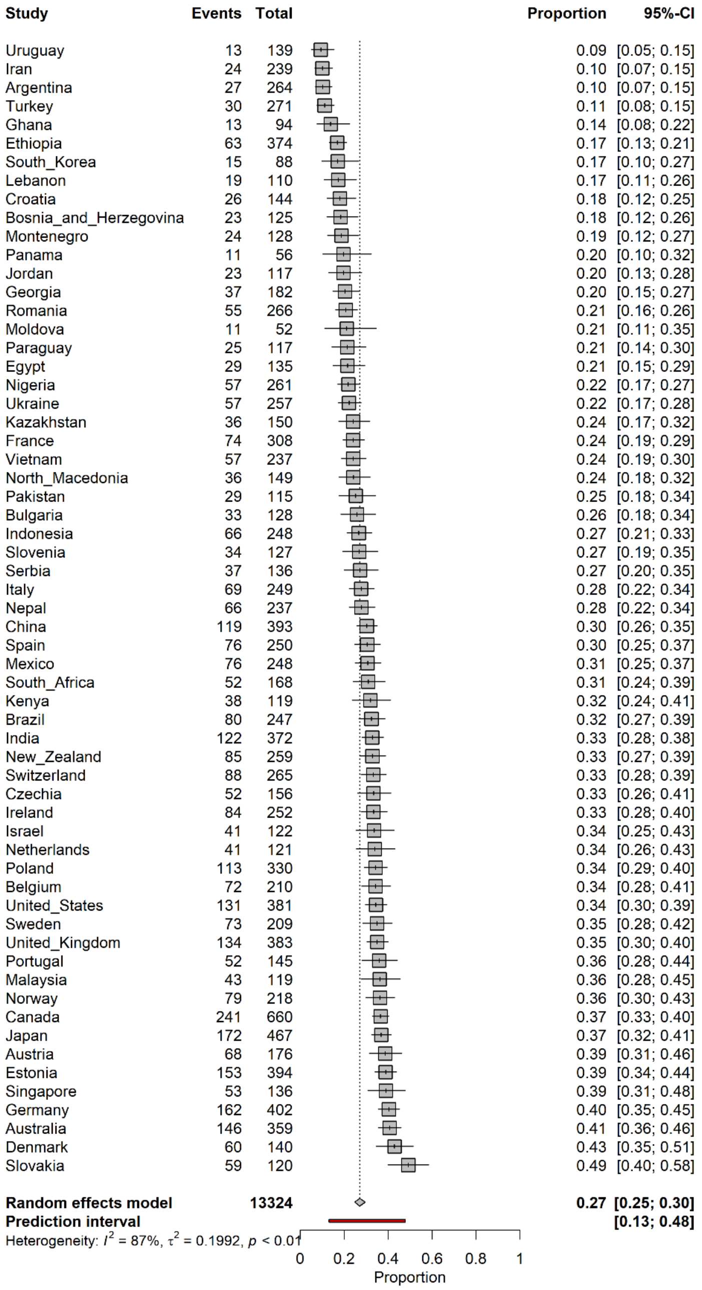

Supplementary Figure 8. Meta-analysis results for subadditivity.

Random effect meta-analysis on the country average temporal discount score. MRAW: sample mean. The overall random effect, prediction interval, and the I^2 Heterogeneity test are presented at the bottom of the figure. The prediction interval is presented in red. A higher proportion indicates a higher rate of engaging in the anomaly.

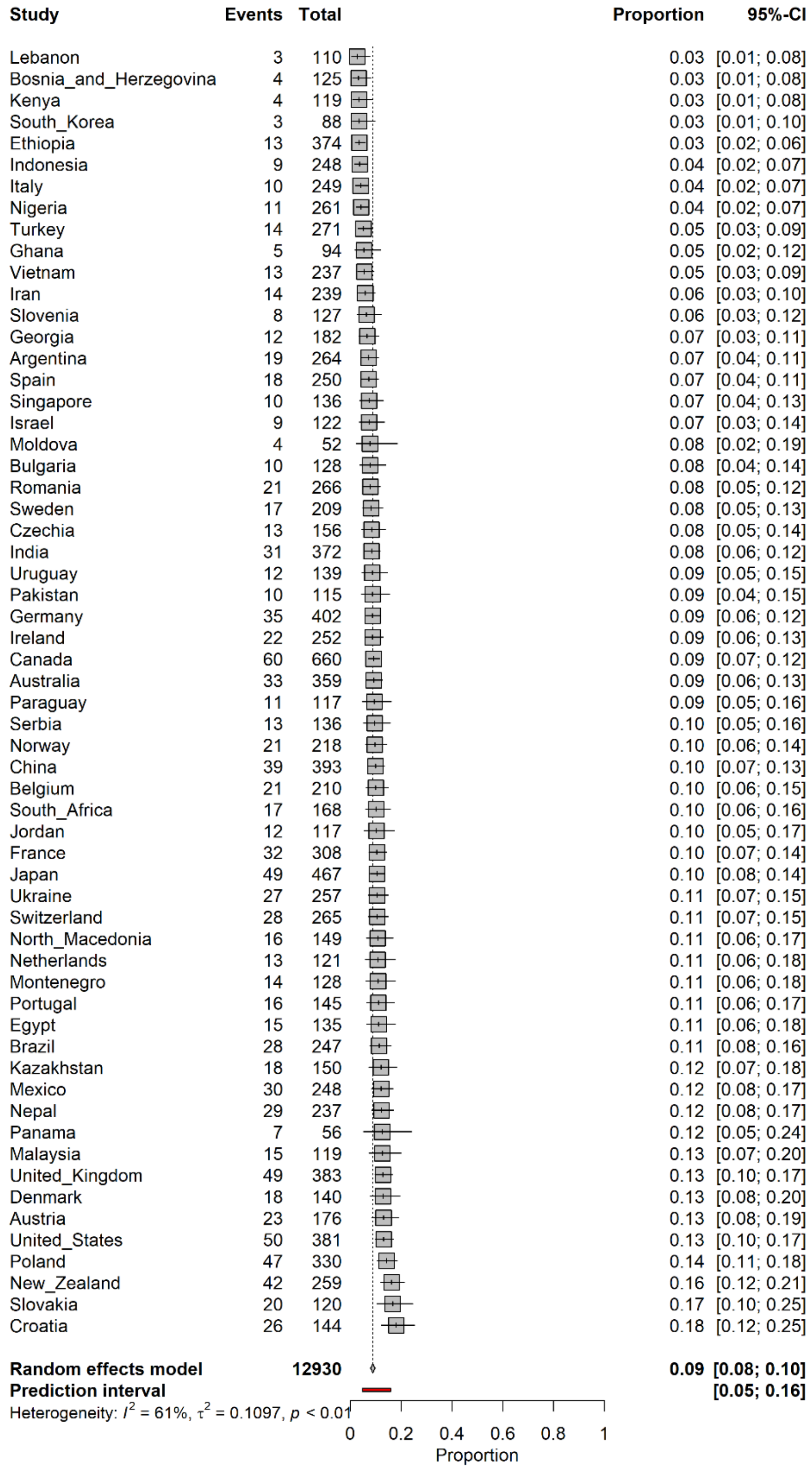

Supplementary Figure 9. Individual and country-level correlation for temporal discount scores and anomalies.

Point-biserial correlation between unstandardized temporal discount scores and anomalies at individual (Panel A) and country-levels (Panel B). P-values were adjusted using the Holm corrections. TDS: Temporal discount score. PB: Present bias. AM: Absolute magnitude. GLS: Gain-loss asymmetry. DS: Delay-speedup. SA: Subadditivity. Blue indicates positive correlations; red color indicates negative correlations. Darker colors reflect the strength of the correlation.

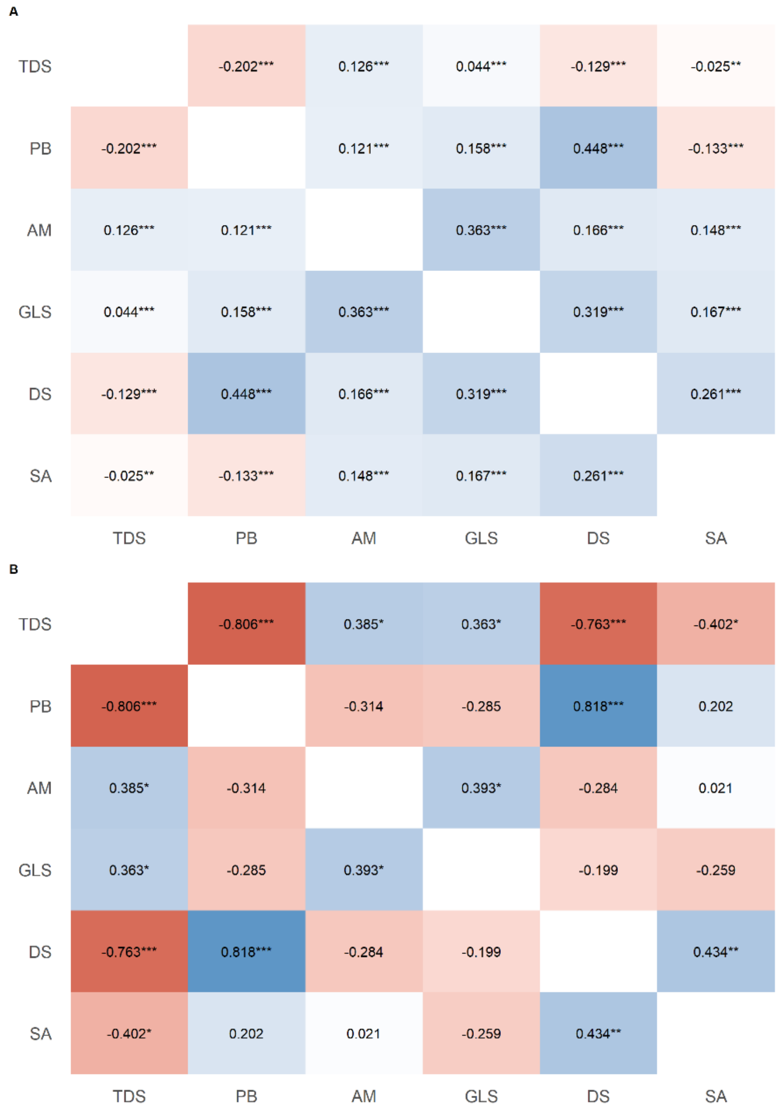

Supplementary Figure 10. Robustness to number of knots on smooth-term estimation

Conditional smooth-effect for inflation, assets, and debt on temporal discount scores controlling for age, education, employment, GDP, and GINI. Positive effect indicates an increasing temporal discounting, and negative effects indicate lower temporal discount effect. We observed no differences in non-linear effects for smooths defined with four knots (top row, A-C) and nine knots (lower row, D-F). Spaghetti plots were employed to assess possible deviation from individual posterior draws. We employed 1000 draws of each posterior distribution of the conditional effects. Average effect depicted in white. Temporal discount scores are standardized, with inflation, debt, and assets being mean-centered per country and scaled.

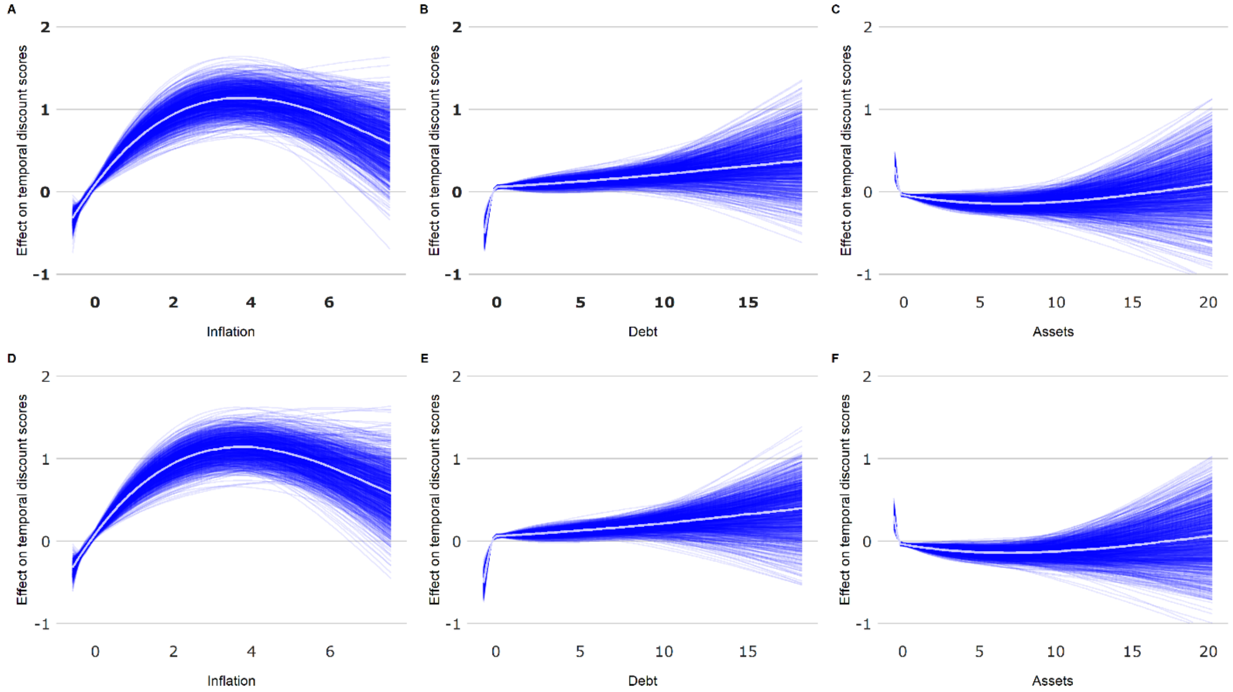

Anomaly	Type	Base value	Delayed options (starting value bold)	Timeline	Notes
**Temporal discounting**	Gain	500	505-510-**550**-600-750	Now vs 12 months	If immediate chosen, next option is increased
**Gain-loss asymmetry**	Loss	500	505-510-**550**-600-750	Now vs 12 months	If immediate chosen, next option is decreased
**Absolute magnitude**	Gain	5000	5050-5010-**5500**-6000-7500	Now vs 12 months	If immediate chosen, next option is increased
**Present bias**	Gain	500	505-510-550-600-750	12 months vs 24 months	Participants only see one delayed option matched to the largest value preferred in temporal discounting set
**Subadditivity**	Gain	500	510-520-600-700-1000	Now vs 24 months	Participants only see one delayed option, which doubles the difference of the largest value preferred in temporal discounting set
**Delay-speedup 1**	Speedup	500	505-510-550-600-750	Now vs 12 months	Single delayed option only. Immediate option framed as a reduction from the larger value for receiving earlier.
**Delay-speedup 2**	Delay	500	505-510-550-600-750	Now vs 12 months	Single delayed option only. Delayed option framed as a bonus value for willing to delay.

Supplementary Table 1. Choice anomaly measures format and content.

Participants responded to 10 to 13 questions, depending on their responses to the initial three sets.

Country	Final sample	Target
Argentina	268	240
Australia	360	240
Austria	178	120
Belgium	215	120
Bosnia and Herzegovina	126	120
Brazil	266	360
Bulgaria	133	120
Canada	665	360
China	398	360
Croatia	146	120
Czechia	162	120
Denmark	144	120
Egypt	139	240
Estonia	394	120
Ethiopia	383	360
France	310	240
Georgia	186	120
Germany	404	240
Ghana	96	240
India	379	360
Indonesia	255	360
Iran	258	240
Ireland	261	120
Israel	125	120
Italy	254	240
Japan	476	360
Jordan	122	120
Kazakhstan	154	240
Kenya	122	240
Lebanon	114	120
Malaysia	123	240
Mexico	258	360
Moldova	55	120
Montenegro	129	120
Nepal	242	240
Netherlands	121	120
New Zealand	264	120
Nigeria	263	360
North Macedonia	157	120
Norway	222	120
Pakistan	123	360
Panama	57	120
Paraguay	121	120
Poland	337	240
Portugal	149	120
Romania	277	240
Serbia	137	120
Singapore	138	120
Slovakia	123	120
Slovenia	128	120

South Africa	174	240
South Korea	93	240
Spain	257	240
Sweden	209	120
Switzerland	269	120
Turkey	276	240
Ukraine	268	240
United Kingdom	388	240
United States	386	360
Uruguay	139	120
Vietnam	253	240

Supplementary Table 2. Sample and target sample size per country.

Final number of participants per country after data exclusions and target data in data collection.

	Age	Gender			Education						Employment					
		Fem.	Male	Oth.	Prim.	Sec.	Tech.	Bach.	Grad.	Stud.	Unem Look.	Unem. P.R.	Empl. P.T.	Empl. F.T.	Self-empl.	Ret.
**Overall**, N = 13,629	34.0 (11.7)	0.47	0.51	0.03	0.01	0.16	0.11	0.40	0.32	0.16	0.04	0.05	0.10	0.53	0.11	0.03
**Argentina**, N = 268	33.5 (12.9)	0.36	0.62	0.02	0.00	0.34	0.09	0.36	0.21	0.19	0.02	0.01	0.08	0.44	0.22	0.04
**Australia**, N = 360	32.7 (9.1)	0.26	0.72	0.02	0.02	0.13	0.14	0.46	0.26	0.09	0.02	0.03	0.07	0.69	0.09	0.02
**Austria**, N = 178	29.3 (8.0)	0.25	0.70	0.05	0.05	0.33	0.15	0.23	0.24	0.21	0.02	0.03	0.16	0.49	0.08	0.01
**Belgium**, N = 215	32.3 (10.2)	0.36	0.57	0.07	0.01	0.18	0.04	0.29	0.48	0.11	0.01	0.02	0.07	0.67	0.10	0.02
**Bosnia and Herzegovina**, N = 126	40.9 (12.1)	0.87	0.10	0.03	0.01	0.06	0.48	0.07	0.38	0.04	0.19	0.20	0.03	0.49	0.00	0.05
**Brazil**, N = 266	34.0 (10.3)	0.54	0.46	0.00	0.00	0.15	0.05	0.24	0.55	0.15	0.06	0.02	0.08	0.46	0.21	0.02
**Bulgaria**, N = 133	36.6 (14.0)	0.53	0.45	0.02	0.02	0.16	0.05	0.26	0.51	0.08	0.02	0.03	0.07	0.65	0.13	0.02
**Canada**, N = 665	33.4 (8.8)	0.33	0.63	0.04	0.01	0.14	0.18	0.46	0.21	0.10	0.02	0.03	0.07	0.71	0.05	0.01
**China**, N = 398	35.2 (10.0)	0.54	0.41	0.06	0.01	0.06	0.26	0.55	0.13	0.09	0.01	0.01	0.08	0.74	0.04	0.03
**Croatia**, N = 146	36.9 (11.1)	0.51	0.48	0.01	0.00	0.29	0.09	0.06	0.57	0.08	0.02	0.03	0.04	0.55	0.24	0.04
**Czechia**, N = 162	29.3 (9.6)	0.59	0.38	0.04	0.04	0.44	0.01	0.17	0.34	0.32	0.05	0.05	0.04	0.44	0.09	0.01
**Denmark**, N = 144	38.1 (15.4)	0.54	0.43	0.03	0.04	0.42	0.16	0.18	0.20	0.23	0.01	0.08	0.07	0.56	0.00	0.05
**Egypt**, N = 139	36.7 (14.9)	0.48	0.48	0.04	0.00	0.09	0.02	0.62	0.27	0.12	0.05	0.05	0.07	0.54	0.11	0.06
**Estonia**, N = 394	36.3 (9.7)	0.80	0.19	0.01	0.01	0.14	0.10	0.32	0.43	0.03	0.02	0.09	0.04	0.71	0.10	0.01
**Ethiopia**, N = 383	29.3 (8.7)	0.29	0.70	0.02	0.05	0.11	0.13	0.57	0.14	0.12	0.19	0.03	0.14	0.38	0.14	0.01
**France**, N = 310	28.1 (9.6)	0.53	0.43	0.04	0.01	0.14	0.02	0.10	0.73	0.36	0.08	0.03	0.11	0.34	0.09	0.00
**Georgia**, N = 186	28.8 (11.6)	0.75	0.23	0.02	0.01	0.09	0.01	0.89	0.00	0.17	0.12	0.09	0.11	0.46	0.04	0.01
**Germany**, N = 404	30.7 (8.7)	0.32	0.62	0.06	0.02	0.29	0.13	0.21	0.34	0.25	0.01	0.03	0.16	0.50	0.04	0.01
**Ghana**, N = 96	31.2 (7.5)	0.24	0.73	0.03	0.00	0.05	0.05	0.78	0.12	0.13	0.12	0.03	0.15	0.45	0.12	0.02

**India**, N = 379	33.3 (13.7)	0.38	0.61	0.02	0.02	0.21	0.00	0.35	0.42	0.19	0.02	0.04	0.12	0.36	0.21	0.05
**Indonesia**, N = 255	32.5 (10.5)	0.53	0.44	0.03	0.02	0.01	0.28	0.46	0.23	0.15	0.04	0.04	0.14	0.49	0.12	0.02
**Iran**, N = 258	31.3 (12.4)	0.54	0.42	0.05	0.01	0.36	0.02	0.37	0.24	0.25	0.07	0.07	0.14	0.30	0.11	0.06
**Ireland**, N = 261	32.4 (9.1)	0.33	0.64	0.03	0.01	0.11	0.09	0.02	0.77	0.14	0.03	0.05	0.05	0.73	0.00	0.01
**Israel**, N = 125	35.2 (11.4)	0.60	0.38	0.02	0.02	0.19	0.03	0.38	0.37	0.23	0.03	0.02	0.11	0.49	0.10	0.02
**Italy**, N = 254	37.6 (13.8)	0.51	0.48	0.02	0.03	0.24	0.06	0.23	0.45	0.23	0.04	0.02	0.07	0.45	0.16	0.03
**Japan**, N = 476	45.0 (11.7)	0.34	0.65	0.01	0.01	0.24	0.16	0.49	0.10	0.06	0.04	0.12	0.12	0.51	0.09	0.06
**Jordan**, N = 122	35.3 (10.7)	0.66	0.26	0.07	0.03	0.14	0.07	0.54	0.22	0.11	0.16	0.11	0.07	0.40	0.12	0.03
**Kazakhstan**, N = 154	28.3 (9.1)	0.67	0.30	0.03	0.00	0.20	0.02	0.50	0.29	0.27	0.03	0.05	0.10	0.48	0.05	0.01
**Kenya**, N = 122	27.7 (6.3)	0.34	0.62	0.03	0.00	0.16	0.32	0.48	0.05	0.19	0.17	0.03	0.25	0.25	0.12	0.00
**Lebanon**, N = 114	25.4 (5.9)	0.53	0.46	0.02	0.00	0.13	0.03	0.55	0.29	0.26	0.12	0.01	0.14	0.35	0.11	0.00
**Malaysia**, N = 123	33.2 (13.1)	0.59	0.39	0.02	0.00	0.08	0.07	0.69	0.16	0.21	0.05	0.07	0.11	0.48	0.07	0.01
**Mexico**, N = 258	30.3 (9.3)	0.19	0.78	0.04	0.00	0.05	0.17	0.60	0.19	0.17	0.05	0.05	0.13	0.59	0.00	0.01
**Moldova**, N = 55	31.4 (12.2)	0.55	0.42	0.04	0.07	0.09	0.06	0.46	0.33	0.15	0.07	0.06	0.07	0.56	0.09	0.00
**Montenegro**, N = 129	41.1 (11.5)	0.66	0.33	0.01	0.00	0.33	0.08	0.28	0.31	0.05	0.06	0.05	0.08	0.54	0.17	0.05
**Nepal**, N = 242	30.1 (10.1)	0.44	0.52	0.04	0.02	0.03	0.23	0.51	0.22	0.28	0.00	0.29	0.14	0.27	0.00	0.01
**Netherlands**, N = 121	38.4 (14.2)	0.56	0.42	0.02	0.01	0.06	0.09	0.33	0.51	0.07	0.03	0.00	0.22	0.53	0.06	0.10
**New Zealand**, N = 264	32.5 (9.8)	0.33	0.63	0.04	0.01	0.17	0.16	0.49	0.17	0.13	0.01	0.05	0.08	0.66	0.06	0.01
**Nigeria**, N = 263	30.3 (7.9)	0.40	0.58	0.02	0.00	0.00	0.31	0.69	0.00	0.48	0.03	0.02	0.11	0.29	0.07	0.00
**North Macedonia**, N = 157	34.7 (8.1)	0.53	0.42	0.05	0.01	0.20	0.05	0.52	0.22	0.03	0.06	0.03	0.05	0.79	0.05	0.00
**Norway**, N = 222	32.2 (10.0)	0.32	0.67	0.01	0.02	0.19	0.08	0.25	0.45	0.24	0.01	0.05	0.05	0.58	0.06	0.00
**Pakistan**, N = 123	29.4 (8.5)	0.49	0.50	0.01	0.03	0.01	0.07	0.57	0.32	0.15	0.05	0.08	0.08	0.46	0.17	0.00
**Panama**, N = 57	40.2 (13.7)	0.54	0.40	0.05	0.00	0.11	0.11	0.35	0.44	0.05	0.04	0.02	0.12	0.54	0.19	0.04

**Paraguay**, N = 121	34.7 (10.5)	0.53	0.45	0.03	0.00	0.06	0.12	0.50	0.33	0.04	0.03	0.02	0.08	0.49	0.33	0.01
**Poland**, N = 337	30.5 (9.5)	0.55	0.44	0.02	0.01	0.26	0.02	0.24	0.47	0.11	0.02	0.02	0.16	0.53	0.14	0.01
**Portugal**, N = 149	36.1 (12.5)	0.54	0.46	0.00	0.01	0.16	0.10	0.42	0.31	0.15	0.07	0.03	0.04	0.66	0.05	0.00
**Romania**, N = 277	36.2 (10.4)	0.70	0.28	0.02	0.01	0.06	0.01	0.36	0.56	0.07	0.03	0.02	0.05	0.70	0.11	0.01
**Serbia**, N = 137	36.3 (10.6)	0.50	0.47	0.03	0.01	0.31	0.06	0.33	0.29	0.12	0.04	0.02	0.05	0.65	0.12	0.01
**Singapore**, N = 138	31.1 (8.4)	0.32	0.65	0.04	0.01	0.01	0.23	0.54	0.23	0.14	0.01	0.00	0.04	0.77	0.04	0.01
**Slovakia**, N = 123	33.9 (12.5)	0.26	0.71	0.03	0.01	0.34	0.04	0.15	0.46	0.20	0.02	0.03	0.03	0.50	0.18	0.04
**Slovenia**, N = 128	37.6 (10.4)	0.41	0.56	0.03	0.01	0.23	0.09	0.26	0.41	0.11	0.01	0.02	0.00	0.69	0.16	0.02
**South Africa**, N = 174	32.7 (10.1)	0.41	0.58	0.02	0.00	0.12	0.06	0.41	0.41	0.21	0.08	0.01	0.11	0.48	0.12	0.00
**South Korea**, N = 93	30.5 (8.9)	0.41	0.58	0.01	0.00	0.04	0.03	0.66	0.26	0.24	0.05	0.11	0.12	0.47	0.00	0.01
**Spain**, N = 257	38.7 (14.8)	0.55	0.42	0.03	0.00	0.03	0.19	0.41	0.38	0.09	0.05	0.05	0.13	0.48	0.09	0.11
**Sweden**, N = 209	37.5 (14.0)	0.59	0.39	0.01	0.05	0.17	0.07	0.30	0.41	0.13	0.02	0.02	0.09	0.61	0.08	0.04
**Switzerland**, N = 269	37.3 (11.0)	0.55	0.43	0.02	0.11	0.21	0.09	0.25	0.35	0.07	0.04	0.12	0.25	0.45	0.05	0.03
**Turkey**, N = 276	44.8 (15.0)	0.39	0.58	0.03	0.01	0.10	0.03	0.63	0.23	0.13	0.03	0.03	0.03	0.32	0.24	0.23
**Ukraine**, N = 268	37.1 (11.5)	0.46	0.52	0.02	0.00	0.03	0.03	0.37	0.57	0.06	0.01	0.02	0.09	0.52	0.27	0.03
**United Kingdom**, N = 388	32.9 (9.7)	0.43	0.54	0.03	0.01	0.09	0.20	0.39	0.32	0.14	0.02	0.06	0.10	0.63	0.06	0.01
**United States**, N = 386	33.3 (12.2)	0.53	0.43	0.04	0.01	0.16	0.05	0.42	0.36	0.25	0.02	0.02	0.09	0.52	0.07	0.03
**Uruguay**, N = 139	47.2 (16.1)	0.48	0.49	0.03	0.01	0.36	0.12	0.39	0.12	0.05	0.04	0.03	0.08	0.35	0.27	0.18
**Vietnam**, N = 253	28.7 (9.2)	0.63	0.33	0.05	0.00	0.30	0.08	0.48	0.14	0.22	0.06	0.03	0.13	0.40	0.17	0.00

Supplementary Table 3. Descriptive statistics for the overall and by country sample.

Descriptive statistics for main individual control variables (age, gender, education, and employment situation) for the overall sample and by country. Mean, and standard deviation was reported for age. The proportion of individuals in each category reported for gender, education, and employment situation. Fem.: Female. Oth.: Other gender. Prim.: Primary education or lower. Sec.:Secondary education. Tech.: Vocational or technical education. Bach.: Bachelor degree. Grad.: Graduate degree. Stud.: Full-time student. Unem. Look.: Unemployed but looking for a job. Unem. P. R.: Unemployed for personal reasons. Empl. P.T.: Employed part-time. Empl. F.T.: Employed full-time, including military services. Self-empl: Self-employed. Ret: Retired. For reference, Supplementary Table 18 provides the reported breakdowns of these demographic within each country.

	Temporal Discount score	Gain	Payment	Large gain	Present Bias	Absolute magnitude	Delay-speedup	Gain-loss assymetry	Subadditivity
**Overall**	10.3 (3.7)	3.6 (1.5)	1.5 (1.8)	2.9 (1.6)	0.15	0.20	0.29	0.39	0.09
Argentina	14.1 (3.0)	4.4 (1.0)	3.6 (1.5)	4.0 (1.2)	0.06	0.19	0.10	0.35	0.07
Australia	9.6 (2.6)	3.3 (1.2)	1.4 (1.4)	2.6 (1.1)	0.26	0.14	0.41	0.29	0.09
Austria	8.1 (2.9)	2.9 (1.5)	0.6 (1.3)	2.1 (1.4)	0.22	0.20	0.39	0.43	0.14
Belgium	8.9 (3.2)	3.2 (1.4)	1.1 (1.6)	2.3 (1.5)	0.19	0.17	0.35	0.32	0.10
Bosnia and Herzegovina	10.5 (3.6)	3.9 (1.6)	1.1 (1.7)	3.4 (1.7)	0.14	0.24	0.19	0.48	0.03
Brazil	10.4 (3.0)	3.6 (1.4)	1.7 (1.5)	2.7 (1.3)	0.15	0.19	0.31	0.31	0.11
Bulgaria	10.9 (3.0)	3.8 (1.4)	1.5 (1.7)	3.3 (1.2)	0.16	0.28	0.26	0.44	0.08
Canada	9.2 (2.9)	3.2 (1.4)	1.2 (1.4)	2.4 (1.2)	0.23	0.16	0.36	0.30	0.09
China	11.4 (3.3)	3.8 (1.4)	2.1 (1.6)	3.3 (1.3)	0.13	0.25	0.30	0.39	0.10
Croatia	9.5 (3.3)	3.6 (1.5)	0.9 (1.6)	2.9 (1.4)	0.09	0.20	0.18	0.43	0.18
Czechia	8.5 (3.1)	3.0 (1.6)	0.8 (1.3)	2.1 (1.5)	0.17	0.22	0.33	0.36	0.08
Denmark	7.8 (3.0)	2.6 (1.5)	0.7 (1.2)	2.1 (1.4)	0.20	0.22	0.43	0.47	0.14
Egypt	12.7 (2.9)	4.3 (1.1)	2.6 (1.8)	3.6 (1.4)	0.05	0.29	0.21	0.43	0.12
Estonia	11.6 (3.1)	3.3 (1.3)	1.1 (1.3)	2.5 (1.3)	/	0.17	0.39	0.35	/
Ethiopia	14.1 (2.9)	4.6 (1.0)	3.0 (2.0)	4.3 (1.2)	0.07	0.24	0.17	0.37	0.03
France	9.9 (3.2)	3.8 (1.3)	1.2 (1.6)	2.6 (1.4)	0.12	0.27	0.24	0.38	0.10
Georgia	11.8 (3.3)	4.5 (1.3)	1.4 (1.9)	3.8 (1.5)	0.14	0.35	0.20	0.62	0.07
Germany	8.0 (2.9)	2.7 (1.4)	0.8 (1.3)	2.0 (1.2)	0.26	0.15	0.40	0.40	0.09
Ghana	13.6 (3.0)	4.6 (1.0)	2.7 (2.1)	4.2 (1.3)	0.05	0.23	0.14	0.49	0.05
India	11.4 (3.0)	4.1 (1.3)	1.4 (1.5)	3.5 (1.4)	0.09	0.26	0.33	0.51	0.08
Indonesia	10.9 (4.6)	3.8 (1.9)	1.4 (2.0)	3.6 (1.9)	0.12	0.13	0.26	0.52	0.04
Iran	13.1 (3.4)	4.4 (1.3)	2.9 (2.0)	3.8 (1.5)	0.07	0.23	0.10	0.43	0.05
Ireland	9.0 (3.0)	3.2 (1.4)	0.9 (1.4)	2.4 (1.3)	0.18	0.15	0.33	0.33	0.08
Israel	7.9 (2.9)	3.0 (1.4)	0.7 (1.2)	1.8 (1.3)	0.23	0.19	0.34	0.35	0.07
Italy	9.4 (3.4)	3.1 (1.6)	1.4 (1.6)	2.4 (1.4)	0.13	0.18	0.28	0.32	0.04
Japan	7.1 (3.9)	2.6 (1.8)	0.6 (1.2)	1.6 (1.6)	0.23	0.21	0.37	0.41	0.11
Jordan	12.2 (2.9)	4.3 (1.2)	2.0 (2.0)	3.6 (1.4)	0.04	0.23	0.19	0.47	0.10
Kazakhstan	11.4 (3.2)	4.2 (1.2)	1.6 (1.9)	3.5 (1.5)	0.07	0.29	0.24	0.49	0.12
Kenya	11.2 (3.7)	4.0 (1.7)	1.7 (1.9)	3.3 (1.8)	0.13	0.22	0.31	0.50	0.04
Lebanon	13.0 (3.0)	4.8 (0.9)	1.6 (2.2)	4.5 (1.3)	0.07	0.09	0.18	0.67	0.03
Malaysia	9.5 (3.5)	3.2 (1.6)	1.3 (1.7)	2.3 (1.6)	0.11	0.20	0.37	0.38	0.14
Mexico	9.8 (3.2)	3.2 (1.5)	1.7 (1.6)	2.4 (1.5)	0.11	0.19	0.31	0.34	0.13
Moldova	10.1 (3.8)	3.8 (1.7)	0.9 (1.4)	3.2 (1.7)	0.06	0.20	0.20	0.46	0.07
Montenegro	11.2 (3.2)	4.1 (1.3)	1.7 (1.9)	3.3 (1.4)	0.10	0.36	0.19	0.44	0.11
Nepal	12.5 (3.8)	4.1 (1.5)	2.4 (2.0)	3.7 (1.6)	0.11	0.25	0.28	0.43	0.13
Netherlands	7.7 (3.0)	2.8 (1.5)	0.5 (1.2)	2.0 (1.5)	0.18	0.15	0.34	0.44	0.11

New Zealand	9.1 (2.8)	3.1 (1.3)	1.2 (1.4)	2.5 (1.3)	0.17	0.13	0.33	0.30	0.16
Nigeria	12.4 (3.6)	4.4 (1.4)	2.0 (2.1)	3.9 (1.6)	0.06	0.19	0.22	0.53	0.04
North Macedonia	10.0 (3.3)	3.7 (1.5)	1.3 (1.7)	2.9 (1.4)	0.13	0.29	0.26	0.41	0.12
Norway	8.4 (2.9)	2.9 (1.5)	0.7 (1.1)	2.4 (1.3)	0.26	0.15	0.37	0.38	0.10
Pakistan	12.0 (2.7)	4.5 (1.0)	1.7 (1.9)	3.6 (1.3)	0.07	0.31	0.25	0.48	0.09
Panama	10.5 (3.5)	3.3 (1.8)	1.8 (1.9)	3.0 (1.7)	0.05	0.12	0.19	0.26	0.12
Paraguay	10.6 (3.6)	3.5 (1.6)	2.0 (1.9)	2.8 (1.6)	0.08	0.21	0.22	0.32	0.09
Poland	9.5 (3.1)	3.4 (1.6)	1.0 (1.5)	2.6 (1.4)	0.15	0.23	0.34	0.42	0.14
Portugal	9.6 (3.6)	3.3 (1.6)	1.3 (1.7)	2.5 (1.6)	0.14	0.22	0.36	0.38	0.11
Romania	10.6 (3.3)	3.9 (1.6)	1.2 (1.7)	3.3 (1.6)	0.09	0.22	0.21	0.47	0.08
Serbia	9.9 (2.9)	3.6 (1.3)	1.1 (1.4)	2.9 (1.2)	0.17	0.28	0.27	0.45	0.10
Singapore	8.2 (3.5)	2.6 (1.4)	1.2 (1.5)	1.9 (1.3)	0.24	0.09	0.39	0.27	0.08
Slovakia	9.1 (3.0)	3.1 (1.6)	1.0 (1.5)	2.5 (1.4)	0.21	0.16	0.50	0.44	0.16
Slovenia	9.2 (3.2)	3.3 (1.4)	1.1 (1.6)	2.6 (1.4)	0.23	0.20	0.27	0.38	0.06
South Africa	10.9 (3.0)	3.8 (1.3)	1.7 (1.7)	3.1 (1.4)	0.16	0.22	0.32	0.28	0.10
South Korea	9.7 (3.1)	3.6 (1.4)	1.2 (1.5)	2.6 (1.4)	0.13	0.16	0.17	0.30	0.03
Spain	9.6 (3.3)	3.4 (1.6)	1.4 (1.7)	2.5 (1.5)	0.18	0.22	0.30	0.35	0.07
Sweden	8.6 (2.7)	3.0 (1.3)	0.6 (1.2)	2.5 (1.3)	0.21	0.18	0.35	0.42	0.08
Switzerland	7.9 (3.8)	2.6 (1.8)	1.1 (1.7)	1.9 (1.6)	0.18	0.17	0.34	0.35	0.10
Turkey	13.8 (2.8)	4.5 (0.9)	3.2 (1.6)	4.1 (1.0)	0.06	0.27	0.11	0.44	0.05
Ukraine	10.9 (3.1)	3.7 (1.4)	1.8 (1.8)	3.1 (1.3)	0.10	0.20	0.23	0.37	0.11
United Kingdom	10.3 (3.7)	3.4 (1.4)	0.9 (1.4)	2.5 (1.3)	0.21	0.21	0.35	0.39	0.13
United States	14.1 (3.0)	3.2 (1.5)	1.2 (1.5)	2.4 (1.5)	0.15	0.18	0.34	0.35	0.13
Uruguay	8.6 (2.6)	3.8 (1.2)	2.1 (1.6)	3.1 (1.2)	0.01	0.13	0.09	0.25	0.09
Vietnam	8.1 (2.9)	3.9 (1.4)	2.0 (1.9)	3.3 (1.5)	0.10	0.24	0.23	0.42	0.05

Supplementary Table 4. Descriptive information for temporal discount scores and anomalies rates per country.

Average (and standard deviation) for total temporal discount score and scores per block (gain, payment, larger gains). The proportion of individuals presenting each anomaly overall and per country. Estonia rates for present bias and subadditivity were missing due to a coding error.

	Temporal discount scores		Present bias		Absolute magnitude		Gain-loss asymmetry		Delay-speedup		Subadditivity	
	**AIC dif**	**p-value**	**AIC dif**	**p-value**	**AIC dif**	**p-value**	**AIC dif**	**p-value**	**AIC dif**	**p-value**	**AIC dif**	**p-value**
**NM**	-	-	-	-	-	-	-	-	-	-	-	-
**NM vs RI**	349.78	<.0001	269.74	<.0001	93.23	<.0001	78.80	**<.0001**	174.05	<.0001	15.80	**.071**
**RI vs RS**	7.06	.079	-4.92	<.0001	-0.01	0.990	-4.40	0.890	-3.52	**.003**	-1.96	**<.0001**

Supplementary Table 5. Model fit comparison for null, random intercept, and random slopes models.

Differences in GAM model fit null models (NM), models including all predictors (age, education, employment, GDP, GINI, individual income inequality, assets, debt, and inflation) country as random intercept (RI) and models including main variables (GINI, individual economic inequality, assets, debt, and inflation) as random slopes (RS). We compared the AIC value and reported p-values from a $\chi 2$ test on fast restricted maximum likelihood scores using corrected degrees of freedom.

	Temporal discount scores		Present bias		Absolute magnitude		Gain-loss asymmetry		Delay-speedup		Subadditivity	
**Fixed estimates**	**Estimate (SD)**	**p-value**	**OR**	**p-value**	**OR**	**p-value**	**OR**	**p-value**	**OR**	**p-value**	**OR**	**p-value**
Intercept	-0.05(0.04)	0.204	0.11	**< 0.0001**	0.27	**< 0.0001**	0.67	**< 0.0001**	0.37	**< 0.0001**	0.09	**< 0.0001**
**Smooth terms**	**Edf**	**p-value**	**Edf**	**p-value**	**Edf**	**p-value**	**Edf**	**p-value**	**Edf**	**p-value**	**Edf**	**p-value**
Econom.Ineq	0.71	0.095	**0.95**	**0.009**	**1.03**	**0.004**	0.66	0.168	0.78	0.081	0.01	0.620
GINI	**1.69**	**0.003**	1.13	0.034	0.01	1.00	2.05	0.020	1.42	0.013	0.01	0.233
Debt	**4.97**	**< 0.0001**	0.91	0.049	0.21	0.273	0.15	0.277	0.73	0.115	0.01	0.720
Assets	**4.96**	**< 0.0001**	**3.04**	**< 0.0001**	**2.63**	**0.001**	0.78	0.041	**3.23**	**< 0.0001**	0.01	0.418
Inflation	**2.55**	**< 0.0001**	**2.51**	**< 0.0001**	**2.26**	**0.001**	**2.88**	**0.0001**	**2.12**	**< 0.0001**	**1.50**	**0.0018**
Residence	**20.99**	**< 0.0001**	**33.98**	**< 0.0001**	**30.29**	**< 0.0001**	**37.48**	**< 0.0001**	**37.44**	**< 0.0001**	**30.01**	**< 0.0001**
Model fit												
Adjusted R^2	0.249		0.023		0.020		0.021		0.041		0.018	

Supplementary Table 6. Effect of main variables on temporal discount scores and anomalies.
Effects estimated using GAM models including country as random intercept and age, gender, education, employment, log GDP as controls. We present standardized estimates for temporal discount scores and odd ratios for anomalies. Scores were standardized, and predictors were standardized and centered at their respective levels. Smooth terms represent non-linear effects, where the edf (effective degrees of freedom) indicate the non-linearity of the effect (being 1 a linear effect, and 0 no effect). The main results are presented bolded and shadowed in grey.

	Temporal discount scores		Present bias		Absolute magnitude		Gain-loss asymmetry		Delay-speedup		Subadditivity	
**Fixed estimates**	**Estimate (SD)**	**p-value**	**OR**	**p-value**	**OR**	**p-value**	**OR**	**p-value**	**OR**	**p-value**	**OR**	**p-value**
Intercept	-0.05(0.04)	0.204	0.11	**< 0.0001**	0.27	**< 0.0001**	0.67	**< 0.0001**	0.37	**< 0.0001**	0.09	**< 0.0001**
**Smooth terms**	**Edf**	**p-value**	**Edf**	**p-value**	**Edf**	**p-value**	**Edf**	**p-value**	**Edf**	**p-value**	**Edf**	**p-value**
Econom.Ineq	0.43	0.18	0.84	0.012	**0.86**	**0.007**	0.66	0.086	0.46	0.171	0.01	0.995
GINI	**0.90**	**0.006**	0.70	0.069	0.01	0.990	0.83	0.021	0.72	0.071	0.01	0.587
Debt	**6.50**	**< 0.0001**	0.81	0.023	0.01	0.950	0.55	0.133	0.74	0.050	0.01	0.551
Assets	**7.18**	**< 0.0001**	**4.97**	**< 0.0001**	**4.48**	**< 0.0001**	0.01	0.413	**6.64**	**< 0.0001**	0.01	0.473
Inflation	**3.09**	**< 0.0001**	**3.10**	**< 0.0001**	**2.56**	**0.003**	**0.91**	**< 0.0001**	**2.47**	**< 0.0001**	**0.91**	**0.001**
Residence	**51.78**	**< 0.0001**	**33.39**	**< 0.0001**	**30.29**	**< 0.0001**	**42.75**	**< 0.0001**	**39.11**	**< 0.0001**	**31.26**	**< 0.0001**
Model fit												
Adjusted R^2	0.251		0.067		0.025		0.021		0.043		0.018	

Supplementary Table 7. Robustness of main results on temporal discount scores and anomalies to change of smooth.

Effects estimated GAM model including country as random intercept and age, gender, education, employment, log GDP as controls. We employed regularized thin-plate splines to estimate non-linear terms. We present standardized estimates for temporal discount scores and odd ratios for anomalies. Scores were standardized, and predictors were standardized and centered at their respective levels. Smooth terms represent non-linear effects, where the Edf (effective degrees of freedom) indicate the non-linearity of the effect (being one a linear effect, and 0 no effect).

Fixed effects	Estimate (95%CI)	p-value
Ind. Econ. Ineq.	-0.01 (-0.03.- 0.001)	0.121
GINI	**0.09 (0.02 – 0.16)**	**0.002**
**Smooth effects**	**Edf**	**p-value**
Inflation	1.81	**< 0.0001**
Debt	2.91	**< 0.0001**
Assets	2.88	**< 0.0001**
**Random effects**		
ICC	0.10	
Marginal R^2 / Conditional R^2	0.037/0.137	

Supplementary Table 8. Effect of main variables on temporal discount scores.

Results from the linear mixed model including country as random intercept and age, gender, education, employment, and log GDP as controls. We present standardized estimates and a 95% confidence interval for fixed effects. Scores were standardized, and predictors were standardized and centered at their respective levels. To facilitate the interpretation, we included a significance test for smooth terms representing non-linear effects from the corresponding GAM model, where the Edf (effective degrees of freedom) indicate the non-linearity of the effect (being one a linear effect, and 0 no effect). ICC represents the intraclass correlation coefficient for country effects. Results significant at 0.01 level are presented bolded and shadowed in grey.

	Present bias		Absolute magnitude		Gain-loss asymmetry		Delay-speedup		Subadditivity	
**Fixed effects**	**OR (95%CI)**	**p-value**	**OR (95%CI)**	**p-value**	**OR (95%CI)**	**p-value**	**OR (95%CI)**	**p-value**	**OR (95%CI)**	**p-value**
Ind. Econ. Ineq.	**1.07 (1.03 -1.13)**	**0.006**	**0.92 (0.87–0.98)**	**0.006**	0.96 (0.92 – 1.00)	0.045	1.04 (1.00 – 1.08)	0.078	1.01 (0.94-1.07)	0.867
GINI	0.92 (0.84 – 1.01)	0.082	1.01 (0.94–1.08)	0.825	0.92 (0.85 – 0.99)	0.023	0.93 (0.87 – 1.01)	0.069	0.98 (0.90-1.07)	0.680
Debt	0.95 (0.90 – 1.00)	0.035	1.00 (0.96-1.05)	0.944	0.97 (0.94 – 1.01)	0.189	0.96 (0.92 -1.00)	0.053	0.98 (0.92–1.05)	0.563
**Smooth effects**	**Edf**	**p-value**	**Edf**	**p-value**	**Edf**	**p-value**	**Edf**	**p-value**	**Edf**	**p-value**
Inflation	**1.63**	**< 0.0001**	**1.92**	**<0.0001**	**1.657**	**0.005**	**1.75**	**< 0.0001**	**1.37**	**0.003**
Assets	**1.01**	**< 0.0001**	**1.96**	**0.009**	0.479	0.144	**2.78**	**< 0.0001**	0.001	0.472
**Random effects**										
ICC	0.03		0.01		0.02		0.02		0.02	
Marginal R^2 / Conditional R^2	0.054/0.082		0.011/0.026		0.010/0.029		0.018/0.037		0.002/0.025	

Supplementary Table 9. Effect of main predictors on anomalies.

Results from generalized mixed model including country as random intercept and age, gender, education, employment, log GDP as controls. We present odds ratios and 95% confidence interval for fixed effects, and predictors were standardized and centered at their respective level. To facilitate the interpretation, we included significance test for smooth terms representing non-linear effects from the corresponding GAM model, where the Edf (effective degrees of freedom) indicate the non-linearity of the effect (being one a linear effect, and 0 no effect). ICC represents the intraclass correlation coefficient for country effects. Results significant at 0.01 level are presented bolded and shadowed in grey.

Fixed effects	Estimate (95%CI)
Ind. Econ. Ineq.	-0.01 (-0.03.- 0.001)
GINI	0.09 (0.02 – 0.17)
**Random effects**	
ICC	0.07
Marginal R^2 / Conditional R^2	0.069/0.247

Supplementary Table 10. Effect of main predictors on temporal discount scores.

Results from Bayesian mixed model including country as random intercept and age, gender, education, employment, and log GDP as controls. Scores were standardized, and predictors were standardized and centered at their respective levels. We present standardized estimates and 95% credible intervals for fixed effects. ICC represents the intraclass correlation coefficient for country effects.

	Present bias	Absolute magnitude	Gain-loss asymmetry	Delay-speedup	Subadditivity
**Fixed effects**	**OR (95%CI)**	**OR (95%CI)**	**OR (95%CI)**	**OR (95%CI)**	**OR (95%CI)**
Ind. Econ. Ineq.	1.08 (1.02 -1.13)	0.92 (0.86 – 0.97)	0.96 (0.92 – 1.00)	1.04 (1.00 – 1.09)	1.01 (0.94-1.08)
GINI	0.92 (0.84 – 1.02)	1.01 (0.94 – 1.09)	0.92 (0.85 – 1.00)	0.93 (0.86 – 1.01)	0.98 (0.90 -1.07)
Debt	0.94 (0.89 – 1.00)	1.00 (0.95 -1.05)	0.98 (0.94 – 1.01)	0.96 (0.92 -1.00)	0.98 (0.92 – 1.05)
**Random effects**					
ICC	0.03	0.01	0.01	0.01	0.02
Marginal R^2 / Conditional R^2	.038/0.054	0.012/0.019	0.013/0.027	0.035/0.045	0.004/0.010

Supplementary Table 11. Effect of main variables on anomalies.

Results from Bayesian generalized mixed models including country as random intercept and age, gender, education, employment, and log GDP as controls. We present odds ratios and 95% credible intervals for fixed effects, and predictors were centered at their respective levels. ICC represents the intraclass correlation coefficient for country effects.

	Temporal Discount Scores		Present bias		Absolute magnitude		Gain-loss asymmetry		Delay-speedup		Subadditivity	
	P	BF_{01}	P	BF_{01}	P	BF_{01}	P	BF_{01}	P	BF_{01}	P	BF_{01}
**Ind. Econ. Ineq.**												
(-0.10 – 0.10)	0.99	>1000	**0.86**	**250**	**0.71**	**91**	0.99	>1000	0.99	>1000	0.99	>1000
(-0.05 – 0.05)	0.98	>1000	**0.19**	**19**	**0.13**	**11**	0.62	125	0.69	200	0.81	333
(-0.01 – 0.01)	0.35	200	**0.09**	**3**	**0.04**	**2**	0.06	24	0.09	34	0.21	100
**GINI**												
(-0.10 – 0.10)	**0.56**	**48**	**0.63**	**59**	0.99	>1000	**0.68**	**77**	0.77	125	0.94	500
(-0.05 – 0.05)	**0.11**	**9**	**0.26**	**26**	0.80	333	**0.18**	**16**	0.29	30	0.66	143
(-0.01 – 0.01)	**0.01**	**3**	**0.06**	**20**	0.20	100	**0.02**	**3**	0.04	15	0.15	77

Supplementary Table 12. Assessment of individual economic inequality and GINI on temporal discount scores and anomalies.

Results presented the proportion of posterior distribution samples that fell within each region of practically equivalence (P) and the log Bayes Factor favoring the null hypothesis (BF_{01}) for each region of equivalence. Posterior distributions were obtained from Bayesian generalized mixed models controlling for age, education, employment, log GDP, assets, and debts. Scores were standardized, and predictors were standardized and centered at their respective levels. BF_{01} between 0 and 0.5 as weak evidence in favor of the null hypothesis, between 0.5 and 1 as moderate evidence in favor of the null effect, and effects larger than one as strong support for the null effect. Main results in dispute (i.e., GINI on temporal discount scores, individual economic inequality on the present bias, absolute magnitude, and delay-speedup) are bolded and grey.

Fixed effects	Estimate (95%CI)	p-value
Ind. Econ. Ineq.	0.01 (0.01.- 0.05)	0.280
GINI	-0.04 (-0.07 – 0.01)	0.081
**Smooth effects**	**Edf**	**p-value**
Inflation	**1.96**	**0.003**
Debt	0.001	0.406
Assets	0.709	0.078
**Random effects**		
ICC	0.01	
Marginal R^2 / Conditional R^2	0.004/0.012	

Supplementary Table 13. Effect of main variables on the sum of anomalies.

Results from the linear mixed model including country as random intercept and age, gender, education, employment, and log GDP as controls. We present standardized estimates and a 95% confidence interval for fixed effects. Scores were standardized, and predictors were standardized and centered at their respective levels. To facilitate the interpretation, we included a significance test for smooth terms representing non-linear effects from the corresponding GAM model, where the Edf (effective degrees of freedom) indicate the non-linearity of the effect (being one a linear effect, and 0 no effect). ICC represents the intraclass correlation coefficient for country effects. Results significant at 0.01 level are presented bolded and shadowed in grey.

	Gain		Payment		Larger gains	
**Fixed estimates**	**Estimate (SD)**	**p-value**	**Estimate (SD)**	**p-value**	**Estimate (SD)**	**p-value**
Intercept	3.59 (0.07)		2.47 (0.09)		2.92 (0.08)	
**Smooth terms**	**Edf**		**Edf**		**Edf**	
Log GDP	1.091	0.0041	0.27	0.248	1.17	< 0.001
**Model fit**						
Adjusted R^2	13.5%		1.08%		14.6%	

Supplementary Table 14. Country-level effects of log GDP on gain, payment, and large gains items.

Differences in GAM model We present unstandardized estimates for gain, payments, and larger gains items. Smooth terms represent non-linear effects, where the Edf (effective degrees of freedom) indicate the non-linearity of the effect (being one a linear effect, and 0 no effect).

	Number of anomalies				
	**0**	**1**	**2**	**3**	**4**
**Overall**	0.46	0.20	0.22	0.09	0.02
**Argentina**	0.55	0.26	0.14	0.04	0.01
**Australia**	0.45	0.14	0.30	0.11	0.01
**Austria**	0.45	0.10	0.28	0.13	0.05
**Belgium**	0.52	0.11	0.21	0.14	0.02
**Bosnia and Herzegovina**	0.44	0.23	0.18	0.12	0.02
**Brazil**	0.47	0.21	0.23	0.09	0.01
**Bulgaria**	0.41	0.20	0.23	0.14	0.01
**Canada**	0.49	0.11	0.28	0.10	0.02
**China**	0.44	0.21	0.24	0.09	0.03
**Croatia**	0.48	0.22	0.24	0.06	0.01
**Czechia**	0.49	0.12	0.22	0.12	0.04
**Denmark**	0.41	0.11	0.30	0.12	0.06
**Egypt**	0.43	0.22	0.30	0.06	0.00
**Ethiopia**	0.45	0.32	0.18	0.06	0.00
**France**	0.48	0.18	0.22	0.09	0.03
**Georgia**	0.27	0.32	0.27	0.11	0.03
**Germany**	0.48	0.09	0.23	0.17	0.04
**Ghana**	0.35	0.46	0.13	0.05	0.01
**India**	0.33	0.25	0.33	0.08	0.01
**Indonesia**	0.39	0.32	0.18	0.11	0.01
**Iran**	0.47	0.29	0.18	0.05	0.01
**Ireland**1	0.52	0.11	0.26	0.09	0.02
**Israel**	0.53	0.06	0.24	0.10	0.06
**Italy**	0.56	0.11	0.22	0.10	0.02
**Japan**	0.53	0.10	0.11	0.17	0.10
**Jordan**	0.43	0.28	0.21	0.07	0.00
**Kazakhstan**	0.40	0.27	0.20	0.12	0.01
**Kenya**	0.38	0.26	0.22	0.10	0.04
**Lebanon**	0.26	0.51	0.19	0.04	0.00
**Malaysia**	0.49	0.14	0.22	0.13	0.02
**Mexico**[1]	0.47	0.21	0.24	0.08	0.01
**Moldova**	0.49	0.20	0.22	0.09	0.00
**Montenegro**,	0.42	0.19	0.30	0.07	0.02
**Nepal**	0.43	0.22	0.23	0.10	0.03
**Netherlands**	0.48	0.15	0.22	0.10	0.06
**New Zealand**,	0.50	0.19	0.21	0.08	0.03
**Nigeria**	0.38	0.35	0.16	0.10	0.01
**North Macedonia**	0.45	0.20	0.20	0.13	0.03
**Norway**	0.46	0.13	0.25	0.13	0.04
**Pakistan**	0.42	0.20	0.23	0.14	0.01
**Panama**	0.56	0.30	0.09	0.05	0.00
**Paraguay**	0.53	0.20	0.20	0.07	0.01
**Poland**	0.45	0.15	0.25	0.12	0.03
**Portugal**	0.45	0.17	0.24	0.11	0.03
**Romania**	0.42	0.26	0.24	0.05	0.02

**Serbia**	0.43	0.15	0.26	0.15	0.02
**Singapore**	0.55	0.09	0.20	0.13	0.03
**Slovakia**	0.35	0.20	0.28	0.15	0.03
**Slovenia**	0.53	0.09	0.21	0.12	0.06
**South Africa**	0.49	0.17	0.22	0.10	0.02
**South Korea**	0.62	0.15	0.12	0.05	0.05
**Spain**	0.48	0.17	0.21	0.09	0.04
**Sweden**	0.49	0.12	0.18	0.17	0.04
**Switzerland**	0.51	0.12	0.24	0.09	0.05
**Turkey**	0.46	0.26	0.22	0.06	0.00
**Ukraine**	0.48	0.22	0.24	0.06	0.00
**United Kingdom**	0.48	0.11	0.21	0.15	0.05
**United States**	0.49	0.16	0.22	0.10	0.03
**Uruguay**	0.66	0.21	0.13	0.01	0.00
**Vietnam**	0.45	0.23	0.22	0.09	0.01

Supplementary Table 15. Descriptive information of the number of anomalies present.

The cumulative number of anomalies for the overall and per country. It is noteworthy that as subadditivity and present bias are mutually exclusive, the former was not considered. Thus, the highest number of anomalies that a participant can show is four. Estonia rates were computed without considering present bias and subadditivity due to a coding error.

	GINI		logGDP		Inflation		Individual economic ineq.		Assets		Debt r/t income	
**Fixed estimates**	**Estimate (SD)**	**p-value**	**Estimate (SD)**	**p-value**	**Estimate (SD)**	**p-value**	**Estimate (SD)**	**p-value**	**Estimate (SD)**	**p-value**	**Estimate (SD)**	**p-value**
Intercept	0.01 (0.05)		0.01 (0.06)		0.01 (0.04)		0.02 (0.05)		0.01 (0.05)		0.01 (0.06)	
**Smooth terms**	**Edf**	**p-value**	**Edf**	**p-value**	**Edf**	**p-value**	**Edf**	**p-value**	**Edf**	**p-value**	**Edf**	**p-value**
	1.456	0.0054	1.027	0.0089	2.43	<0.001	0.001	0.736	1.91	0.0002	0.001	0.589
Model fit												
Adjusted R^2	13.7%		11.2%		56.3%		0.001%		25.7%		0.001%	

Supplementary Table 16. Non-linear relationship between each variable and country-aggregated temporal discount scores.

GAM model at country level predicting standardized temporal discount scores by each relevant predictor. Smooth terms represent non-linear effects, where the Edf (effective degrees of freedom) indicate the non-linearity of the effect (being one a linear effect, and 0 no effect).

	Temporal discount scores		Present bias		Absolute magnitude		Gain-loss asymmetry		Delay-speedup		Subadditivity	
**Fixed estimates**	**Estimate (SD)**	**p-value**	**OR**	**p-value**	**OR**	**p-value**	**OR**	**p-value**	**OR**	**p-value**	**OR**	**p-value**
Intercept	-0.05(0.04)	0.204	0.11	**< 0.0001**	0.27	**< 0.0001**	0.67	**< 0.0001**	0.37	**< 0.0001**	0.09	**< 0.0001**
**Smooth terms**	**Edf**	**p-value**	**Edf**	**p-value**	**Edf**	**p-value**	**Edf**	**p-value**	**Edf**	**p-value**	**Edf**	**p-value**
Econom.Ineq	0.15	0.279	0.82	0.076	0.92	0.042	0.19	0.265	0.38	0.212	0.01	0.447
GINI	**1.66**	**0.003**	1.05	0.038	0.01	0.942	2.09	0.018	1.30	0.019	0.01	0.404
Debt	**5.71**	**< 0.0001**	0.98	0.038	0.34	0.222	0.11	0.290	0.55	0.145	0.01	0.995
Assets	**5.31**	**< 0.0001**	**2.96**	**< 0.0001**	**2.27**	**0.002**	0.86	0.015	**3.17**	**< 0.0001**	0.01	0.732
Inflation	**2.61**	**< 0.0001**	**2.57**	**< 0.0001**	**2.67**	**0.002**	**3.02**	**0.001**	**1.99**	**< 0.0001**	**1.53**	**0.001**
Residence	**50.92**	**< 0.0001**	**33.20**	**< 0.0001**	**27.71**	**< 0.0001**	**36.03**	**< 0.0001**	**37.77**	**< 0.0001**	**30.02**	**< 0.0001**
Model fit												
Adjusted R^2	0.252		0.066		0.019		0.021		0.041		0.018	

Supplementary Table 17. Sensitivity analysis of main variables on temporal discount scores and anomalies.
Effects estimated using GAM models including country as random intercept and age, gender, education, employment, log GDP as controls. We present standardized estimates for temporal discount scores and odd ratios for anomalies. Scores were standardized, and predictors were standardized and centered at their respective levels. Smooth terms represent non-linear effects, where the edf (effective degrees of freedom) indicate the non-linearity of the effect (being 1 a linear effect, and 0 no effect). The main results are presented bolded and shadowed in grey. Data was filtered using more conservative criterions for assets and income.

Country	% Population, age dependency ratio (2020)[1]	% Population, female (2020)[2]	Unemployment rate (2020)[3]	% Population, ages 25-64, with post secondary education attainment	Year	% Population Retired (based on country-level eligibility)	Year	Notes
**Argentina**	55.77	51.21	11.67	27.98	2020[4]	11.20	2019[29]	29. % over 65
**Australia**	55.05	50.20	6.61	34.40	2020[4]	16.70	2021[30]	
**Austria**	50.64	50.71	5.77	51.50	2020[4]	19.50	2022[31]	31. Percentage of people aged 65 and older; retirement age is 65 for men and 60 for women
**Belgium**	56.96	50.44	6.01	37.30	2020[4]	19.00	2019[29]	29. % over 65
**Bosnia_and_Herzegovina**	48.01	51.03	16.85	13.70	2020[5]	17.20	2019[29]	29. % over 65
**Brazil**	43.48	50.87	13.67	20.10	2020[4]	9.30	2019[29]	29. % over 65
**Bulgaria**	56.61	51.44	5.71	29.20	2020[6]	21.30	2019[29]	29. % over 65
**Canada**	51.24	50.37	9.48	32.50	2020[4]	16.90	2016[32]	
**China**	42.21	48.71	5.00	9.70	2020[7]	11.50	2019[29]	29. % over 65
**Croatia**	55.75	51.79	7.20	36.60	2020[8]	21.30	2020[33]	33. Calculated by dividing the number of people above 65 years old by total 2021 population
**Czechia**	56.00	50.77	2.94	69.20	2020[4]	22.30	2021[34]	34. Calculated by dividing the current number of pensioners by total 2021 population
**Denmark**	57.35	50.29	5.66	38.00	2020[5]	20.00	2019[29]	29. % over 65
**Egypt**	64.62	49.48	10.45	13.00	2017[5]	8.03	2021[35]	35. Summed up the total percentage of population with retirement age above 60 years old
**Estonia**	58.41	52.62	6.46	48.40	2020[4]	20.00	2019[29]	29. % over 65
**Ethiopia**	76.85	49.97	2.79	10.40	2018[9]	3.50	2019[29]	29. % over 65
**France**	62.36	51.60	8.62	41.80	2020[4]	25.90	2019[36]	
**Georgia**	54.98	52.34	12.05	30.00	2020[10]	21.29	2021[37]	
**Germany**	55.38	50.57	4.31	54.90	2020[4]	22.00	2020[38, 39]	
**Ghana**	67.42	49.31	4.53	18.70	2020[11]	3.10	2019[29]	29. % over 65
**India**	48.66	48.04	7.11	29.00	2019[12]	22.70	2020[40]	
**Indonesia**	47.49	49.65	4.11	28.60	2020[4]	6.10	2019[29]	29. % over 65
**Iran**	45.58	49.51	10.96	16.10	2018[13]	11.30	2016[41]	41. Retirement age is 60 for men and 55 for women
**Ireland**	54.82	50.36	5.92	35.50	2020[4]	14.20	2019[29]	29. % over 65
**Israel**	67.34	50.23	4.61	37.90	2020[4]	12.10	2020[42]	
**Italy**	56.96	51.31	9.31	42.70	2020[4]	26.89	2021[43]	
**Japan**	69.05	51.17	2.97	52.70	2020[14]	28.00	2019[29]	29. % over 65
**Jordan**	58.25	49.37	18.50	32.60	2020[5]	3.90	2019[29]	29. % over 65
**Kazakhstan**	58.85	51.47	6.05	79.00	2018[5]	7.70	2019[29]	29. % over 65
**Kenya**	69.78	50.31	2.98	10.00	2019[15]	2.40	2019[29]	29. % over 65
**Lebanon**	48.41	49.66	6.61	28.3-33.9	2019[16]	7.30	2019[29]	29. National education data separates women (left) and men (right): Age% over 65
**Malaysia**	44.16	48.62	4.55	22.55	2019[17]	7.40	2021[44]	
**Mexico**	50.27	51.08	4.71	22.20	2020[4]	3.20	2017[45]	
**Moldova**	39.63	52.11	4.71	58.00	2020[18]	12.00	2019[29]	29. % over 65
**Montenegro**	51.09	50.55	15.86	17.00	2011[19]	18.49	2021[46]	
**Nepal**	52.99	54.19	4.44	9.30	2011[20]	6.00	2020[47]	
**Netherlands**	55.61	50.18	4.09	38.40	2020[4]	19.60	2019[29]	29. % over 65
**New_Zealand**	55.76	50.85	4.55	40.80	2020[4]	16.40	2020[48]	
**Nigeria**	85.96	49.32	9.01	16.80	2020[21]	2.70	2019[29]	29. % over 65
**North_Macedonia**	44.48	49.98	18.40	22.00	2020[5]	14.10	2019[29]	29. % over 65
**Norway**	53.34	49.46	4.62	37.20	2020[4]	17.20	2020[49]	
**Pakistan**	64.39	48.54	4.65	12.20	2019[22]	3.80	2020[50]	50. Developed a custom indicator for retirement in world bank. The % of female pop above 55 yrs plus % of male population above 60 yrs. As these are the set retirement ages in Pakistan and this is as close a statistic to finding out, % of population that is retired.
**Panama**	53.93	49.94	10.23	47.80	2016[23]	8.30	2019[29]	29. % over 65
**Paraguay**	55.53	49.19	7.61	**N/A**	**N/A**	6.60	2019[29]	29. % over 65
**Poland**	51.42	51.55	3.55	60.40	2020[4]	18.10	2019[29]	29. % over 65
**Portugal**	55.83	52.69	7.20	27.20	2020[4]	22.40	2019[29]	29. % over 65
**Romania**	53.26	51.38	4.84	18.40	2019[5]	18.80	2019[29]	29. % over 65
**Serbia**	52.50	51.02	9.08	27.90	2011[24]	24.60	2020[51]	51. Educational attainment data is quite old with no alternative
**Singapore**	34.50	47.66	5.19	61.80	2021[25]	11.70	2021[52]	52. Retirement age is 62, but population statistics only provide the percentage of those over 65 years old, i.e., 16.0%
**Slovakia**	47.61	51.31	6.79	65.70	2020[4]	25.80	2021[53]	53. Retirement age eligibility varies, there were 1 407 596 eligible people in December 2021 (see the reference), population is 5 449 270 based on a census from the same year
**Slovenia**	55.94	50.20	5.17	54.40	2020[4]	29.30	2021[54]	
**South Africa**	52.23	50.74	28.74	31.80	2020[4]	5.40	2019[29]	29. % over 65
**South Korea**	39.54	49.94	4.07	38.60	2020[4]	16.50	2021[55]	55. % over 65
**Spain**	52.39	50.85	15.67	23.20	2020[4]	19.60	2019[29]	29. % over 65
**Sweden**	61.17	49.91	8.45	39.30	2020[4]	21.90	2021[56]	
**Switzerland**	51.64	50.39	4.94	44.00	2020[4]	19.40	2020[57]	57. Retirement age eligibility varies between men (65) and women (64)
**Turkey**	49.08	50.63	13.92	19.75	2020[4]	8.70	2019[29]	29. % over 65
**Ukraine**	49.12	53.67	9.48	65.00	2020[26]	25.68	2019[58]	58. Gives 80%, the recent academic report gives 78%. Note that official reports do NOT have this information. Based on avaliable raw data that we have on national demographics, the more accurate estimate is 64%. Thus, the more realistic one is 64%, the one that can be backed up by national stats is 80%.
**United Kingdom**	57.06	50.59	4.34	32.30	2020[4]	18.50	2019[29]	29. % over 65
**United States**	53.85	50.52	8.31	41.67	2020[4]	19.30	2021[59]	
**Uruguay**	54.85	51.68	12.67	25.10	2019[27]	14.90	2019[29]	29. % over 65
**Vietnam**	45.05	50.07	2.27	28.60	2019[28]	7.60	2019[29]	29. % over 65

Supplementary Table 18. Compilation of national demographic profiles for general reference. These data are compiled from a large number of varying sources, often with different definitions and methodologies. The compilation is entirely for the purpose of comparing to this study sample and we urge some caution in using this as a primary reference.

1. The World Bank (2022). *Age dependency ratio (% of working-age population).* url: https://data.worldbank.org/indicator/SP.POP.DPND.
2. The World Bank (2022). *Population, female (% of total population).* url: https://data.worldbank.org/indicator/SP.POP.TOTL.FE.ZS.
3. The World Bank (2022). *Unemployment, total (% of total labor force) (national estimate).* url: https://data.worldbank.org/indicator/SL.UEM.TOTL.NE.ZS.
4. OECD (2022). *Adult education level* (indicator). doi: 10.1787/36bce3fe-en (Accessed on 23 February 2022)
5. The World Bank (2022). *Educational Attainment, at least completed post-secondary, population 25+* . Url: https://data.worldbank.org/indicator/SE.SEC.CUAT.PO.ZS?end=2020&most_recent_value_desc=true&start=1970&view=chart
6. Republic of Bulgaria National Statistical Institute (2022). *Total.* url: https://nsi.bg/en/content/14772/total
7. OECD (2022). *China Overview of the education system.* url: https://gpseducation.oecd.org/CountryProfile?primaryCountry=CHN&treshold=10&topic=EO
8. European Commission (2022). *Education and Training Monitor 2021, Croatia* . url: https://op.europa.eu/webpub/eac/education-and-training-monitor-2021/en/croatia.html
9. UNESCO Institute for Statistics (2022). *Ethiopia* . url: http://uis.unesco.org/country/ET
10. Caucasus Research Resource Center (2022). RESPEDU: Highest level of education you have achieved. Url: https://caucasusbarometer.org/en/cb2020ge/RESPEDU-withoutdkra/
11. UNESCO Institude for Statistics (2022). *Ghana* . Url:http://uis.unesco.org/country/GH
12. OECD (2022). *Education at a glance 2019* . url: https://www.oecd.org/education/education-at-a-glance/EAG2019_CN_IND.pdf
13. https://www.amar.org.ir/Portals/0/PropertyAgent/461/Files/9246/0100O99Z.xlsx Url: . جدول- 10 جمعيت 10 ساله وبيشتربرحسب وضع سواد، آخرين مدرك يادوره تحصيلي باسوادان به تفكيك جنس و وضع فعاليت اقتصادئ (2022). Iran of Center Statistical 13.
14. OECD (2022). *Japan Overview of the education system*. Url: https://gpseducation.oecd.org/CountryProfile?primaryCountry=JPN&treshold=10&topic=EO
15. UNESCO Institute for Statistics (2022). *Kenya* . Url: http://uis.unesco.org/country/KE
16. UNDP (2022). *Educational attainment of women and men aged 25 years and above, by age bracket, 2004, 2007, and 2018-2019* . url: http://www.cas.gov.lb/images/Publications/Gender/GENDER%20REPORT.pdf
17. The World Bank (2022). *Educational Attainment, at least completed post-secondary, population 25+* . Url: https://data.worldbank.org/indicator/SE.SEC.CUAT.PO.ZS?page=6&locations=MY
18. UNESCO Institute for Statistics (2022). *Republic of Moldova* . Url: http://uis.unesco.org/country/MD
19. CRNA GORAZAVOD ZA STATISTIKUS A O P Š T E NJ E (MONSTAT) (2022). *Obrazovna struktura stanovništva Crne Gore.* Url: http://monstat.org/userfiles/file/popis2011/Saop_obraz%2014_06_2012_%20konacno%20PDF.pdf
20. The World Bank (2022). *Educational Attainment, at least completed post-secondary, population 25+ - Nepal*. Url: https://data.worldbank.org/indicator/SE.SEC.CUAT.PO.ZS?end=2011&locations=NP&page=6&start=1971&view=chart
21. National Bureau of Statistics (2022). *NYS Report 2020* . url: https://nigerianstat.gov.ng/download/1241079
22. UNESCO Institute for Statistics (2022). *Pakistan* . Url: http://uis.unesco.org/en/country/pk
23. UNESCO Institute for Statistics (2022). *Panama.* Url: http://uis.unesco.org/country/PA
24. Републички завод за статистику (2022). ПОПУЛАЦИЈА СРБИЈЕ ПОЧЕТКОМ *21. ВЕКА.* Url: https://publikacije.stat.gov.rs/G2015/Pdf/G20154006.pdf
25. Department of Statistics Singapore (2022). *Education, Language Spoken and Literacy.* Url: https://www.singstat.gov.sg/find-data/search-by-theme/population/education-language-spoken-and-literacy/latest-data
26. Ministry of Education and Science of Ukraine (2022). *Ozvitku vishchoi osviti v ukraini 02-10-2020* . url: https://mon.gov.ua/storage/app/media/rizne/2020/09/25/rozvitku-vishchoi-osviti-v-ukraini-02-10-2020.pdf
27. Instituto Nacional de Estadística Instituto Nacional de Estadística Uruguay (2022). Url: https://www.ine.gub.uy/educacion
28. UNESCO Institute for Statistics (2022). *Viet Nam*. Url: http://uis.unesco.org/country/VN
29. United Nations Department of Economic and Social Affairs (2022). *World Population Ageing 2019* . url: https://www.un.org/en/development/desa/population/publications/pdf/ageing/WorldPopulationAgeing2019-Highlights.pdf
30. Australian Institute of Health and Welfare (2022). *Age Pension Recipients, June 2001 to June 2021.* url: https://www.aihw.gov.au/reports/australias-welfare/age-pension
31. STATISTIK AUSTRIA, Letzte Änderung am (2022). *Bevölkerung nach Alter und Geschlecht* . Url: https://www.statistik.at/web_de/statistiken/menschen_und_gesellschaft/bevoelkerung/bevoelkerungsstruktur/bevoelkerung_nach_alter_geschlecht/index.html
32. Statistics Canada (2022). *Census Profile, 2016 Census.* Url: https://www12.statcan.gc.ca/census-recensement/2016/dp-pd/prof/details/page.cfm?Lang=E&Geo1=PR&Code1=01&Geo2=PR&Code2=01&SearchText=Canada&SearchType=Begins&SearchPR=01&B1=Population&TABID=1&type=1
33. Republic of Croatia - Croatian Bureau of Statistics (2022). *Registered unemployment rate in the Republic of Croatia* . Url: https://www.dzs.hr/default_e.htm
34. Česká správa sociálního zabezpečení (2022). Důchodová statistika. Url: https://www.cssz.cz/duchodova-statistika
35. Statista (2022). *Total population of Egypt as of 2021, by age group.* Url: https://www.statista.com/statistics/1230371/total-population-of-egypt-by-age-group/
36. Direction de la recherche, des études de l'évaluation et des statistiques (2022). *Vue d'ensemble Fiches thématiques Annexes.* Url: https://drees.solidarites-sante.gouv.fr/sites/default/files/2021-01/Vue%20d%27ensemble_2.pdf
37. საქსტატის ოფიციალური ვებგვერდი (2022). სოციალური უზრუნველყოფა. Url: https://www.geostat.ge/ka/modules/categories/55/sotsialuri-uzrunvelqofa
38. Statistisches Bundesamt (Destatis) (2022). Bevölkerung Ältere Menschen. Url: https://www.destatis.de/DE/Themen/Querschnitt/Demografischer-Wandel/Aeltere-Menschen/bevoelkerung-ab-65-j.html
39. Statista (2022). *Senioren in Deutschland – Statistiken und Daten.* Url: https://de.statista.com/themen/172/senioren/#dossierKeyfigures
40. OECD (2022). *Working age population* . Url: https://data.oecd.org/pop/working-age-population.htm
41. Statistical Centre of Iran (2022). *Selected Findings of the 2016 National Population and Housing Census* . Url: https://www.amar.org.ir/Portals/1/census/2016/Census_2016_Selected_Findings.pdf
42. Central Bureau of Statistics (2022). *International Senior Citizen's Day 2021: Selected Data on Various Topics Regarding Israeli Senior Citizens Aged 65 and Over.* Url: https://www.cbs.gov.il/he/mediarelease/DocLib/2021/321/11_21_321e.pdf
43. Istituto Nazionale di Statistica (Istat) (2022). *QUEI 16 MILIONI DI PENSIONATI IN ITALIA* . Url: https://www.istat.it/it/archivio/254290
44. DOSM (2022). *Current Population Estimates, Malaysia, 2021* . url: https://www.dosm.gov.my/v1/index.php?r=column/cthemeByCat&cat=155&bul_id=ZjJOSnpJR21sQWVUcUp6ODRudm5JZz09&menu_id=L0pheU43NWJwRWVSZklWdzQ4TlhUUT09
45. INEGI (2022). *Encuesta Nacional de Empleo y Seguridad Social (ENESS) 2017* . url: https://www.inegi.org.mx/programas/eness/2017/#Microdatos
46. Uprava za statistiku Crne Gore - MONSTAT (2022). *Crna Gora u brojkama 2021* . url: https://monstat.org/uploads/files/publikacije/Monstat%20-%20CG%20u%20Brojkama%20MNE_WEB.pdf
47. The World Bank (2022). *Population ages 65 and above (% of total population) - Nepal.* url: https://data.worldbank.org/indicator/SP.POP.65UP.TO.ZS?locations=NP
48. The World Bank (2022). *Population ages 65 and above (% of total population) - New Zealand* . Url: https://data.worldbank.org/indicator/SP.POP.65UP.TO.ZS?locations=NZ
49. Statistics Norway (2022). *Alderspensjonister, etter kjønn og alder.* Url: https://www.ssb.no/sosiale-forhold-og-kriminalitet/trygd-og-stonad/statistikk/alderspensjonister
50. The World Bank (2022). https://databank.worldbank.org/source/health-nutrition-and-population-statistics
51. Republički Fond za penzijsko i invalidsko osiguranje, Direkcija (2022). *СТАТИСТИЧКИ ГОДИШЊИ БИЛТЕН 2020.* url: https://www.pio.rs/sites/default/files/Statistike/Bilten/Statisticki%20godisnji%20bilten%20za%202020.pdf
52. Department fo Statistics Signapore (2022). *Elderly, Youth and Gender Profile.* Url: https://www.singstat.gov.sg/find-data/search-by-theme/population/elderly-youth-and-gender-profile/latest-data
53. Sociálna poisťovňa, ústredie, Ul (2022). *Počet vyplácaných dôchodkov (v mesiacoch)*. Url: https://www.socpoist.sk/pocet-vyplacanych-dochodkov--v-mesiacoch-/3150s
54. ZPIZ - Zavod za pokojninsko in invalidsko zavarovanje (2022). *UŽIVALCI POKOJNIN IZ OBVEZNEGA ZAVAROVANJA IN UŽIVALCI DELA STAROSTNE ALI PREDČASNE POKOJNINE, 12/2021* . url: https://www.zpiz.si/wps/portal/cognos/msp/upokojenci/prejemniki_pokojnin
55. Statistics Korea (2022). *Share of the elderly population aged 65 and over*. Url: http://kostat.go.kr/assist/synap/preview/skin/miri.html?fn=d260e147732685916112421&rs=/assist/synap/preview
56. Statistikdatabas Pensionsstatistik (2022). *Allmän pension och inkomstpensionstillägg - månadsutbetalningar*. Url: https://www.pensionsmyndigheten.se/statistik/pensionsstatistik/?domain=tab-3&report=report-3-1&columns=APIPTTot&rows=aldersklasser&sex=Samtliga&metrics=Antal&Folkbokford=Samtliga&time=2021-11-01&complete-input=
57. Swiss Federal Statistical Office (SFSO) (2022). *Permanent resident population by age, sex and category of citizenship, 2010-2020.* url: https://www.bfs.admin.ch/bfs/en/home/statistics/population/effectif-change/age-marital-status-nationality.assetdetail.18344213.html
58. Пенсійний фонд України (2022). Chyselnist pensioneriv ta serednij rozmir yih pensij stanom na 01-04-2019. url: https://www.pfu.gov.ua/1617635-chyselnist-pensioneriv-ta-serednij-rozmir-yih-pensij-stanom-na-01-04-2019/
59. Miguel Faria e Castro, "The COVID Retirement Boom," Economic Synopses, No. 25, 2021. https://doi.org/10.20955/es.2021.25

References

1. Ruggeri, K. *et al.* Replicating patterns of prospect theory for decision under risk. *Nat Hum Behav* **4**, 622–633 (2020).
2. Cavagnaro, D. R., Aranovich, G. J., McClure, S. M., Pitt, M. A. & Myung, J. I. On the functional form of temporal discounting: An optimized adaptive test. *J Risk Uncertain* **52**, 233–254 (2016).
3. Dai, J. & Busemeyer, J. R. A probabilistic, dynamic, and attribute-wise model of intertemporal choice. *J Exp Psychol Gen* **143**, 1489–1514 (2014).
4. Molloy, M. F. *et al.* Hierarchies improve individual assessment of temporal discounting behavior. *Decision (Wash D C)* **7**, 212–224 (2020).
5. Bailey, A. J., Romeu, R. J. & Finn, P. R. The problems with delay discounting: a critical review of current practices and clinical applications. *Psychol Med* **51**, 1799–1806 (2021).
