# Supplementary material for: The globalizability of temporal discounting
Source: Nat Hum Behav. 2022 Jul 11;6(10):1386–97. doi: 10.1038/s41562-022-01392-w (PMC9584811; doi:10.1038/s41562-022-01392-w)
Supplement: Supplementary file 2 — Reporting Summary [file 41562_2022_1392_MOESM2_ESM.pdf]

nature portfolio

Corresponding author(s): Kai Ruggeri

Last updated by author(s): Apr 6, 2022

Reporting Summary

Nature Portfolio wishes to improve the reproducibility of the work that we publish. This form provides structure for consistency and transparency in reporting. For further information on Nature Portfolio policies, see our Editorial Policies and the Editorial Policy Checklist.

Statistics

For all statistical analyses, confirm that the following items are present in the figure legend, table legend, main text, or Methods section.

n/a	Confirmed	
☐	☒	The exact sample size (*n*) for each experimental group/condition, given as a discrete number and unit of measurement
☐	☒	A statement on whether measurements were taken from distinct samples or whether the same sample was measured repeatedly
☐	☒	The statistical test(s) used AND whether they are one- or two-sided<br>*Only common tests should be described solely by name; describe more complex techniques in the Methods section.*
☐	☒	A description of all covariates tested
☐	☒	A description of any assumptions or corrections, such as tests of normality and adjustment for multiple comparisons
☐	☒	A full description of the statistical parameters including central tendency (e.g. means) or other basic estimates (e.g. regression coefficient) AND variation (e.g. standard deviation) or associated estimates of uncertainty (e.g. confidence intervals)
☐	☒	For null hypothesis testing, the test statistic (e.g. *F*, *t*, *r*) with confidence intervals, effect sizes, degrees of freedom and *P* value noted<br>*Give P values as exact values whenever suitable.*
☐	☒	For Bayesian analysis, information on the choice of priors and Markov chain Monte Carlo settings
☐	☒	For hierarchical and complex designs, identification of the appropriate level for tests and full reporting of outcomes
☐	☒	Estimates of effect sizes (e.g. Cohen's *d*, Pearson's *r*), indicating how they were calculated

Our web collection on statistics for biologists contains articles on many of the points above.

Software and code

Policy information about availability of computer code

Data collection	Data collection was conducted using the Qualtrics XM web service platform.
Data analysis	All code relative to power estimation and pre-registration is openly available in https://osf.io/jfvh4 with all data stored at https://osf.io/njd62/. All analyses were conducted in R 4.0.2 using the Microsoft R Open Distribution 4.0.2. Principal packages employed were tidyverse (1.3.0) for data handling, meta (4.18-2) for estimating meta-analyses, mgcv (1.8-31) for estimating hierarchical generalized additive models, gamm4 (0.2-6) for estimating mixed linear and generalized models, and brms (2.14.4) for computing the Bayesian version of the latter. All visualizations were created using ggplot2 (3.3.3.). Analysis code and data will be publicly prior to publication in the same link.

For manuscripts utilizing custom algorithms or software that are central to the research but not yet described in published literature, software must be made available to editors and reviewers. We strongly encourage code deposition in a community repository (e.g. GitHub). See the Nature Portfolio guidelines for submitting code & software for further information.

Data

Policy information about availability of data

All manuscripts must include a data availability statement. This statement should provide the following information, where applicable:

- Accession codes, unique identifiers, or web links for publicly available datasets
- A description of any restrictions on data availability
- For clinical datasets or third party data, please ensure that the statement adheres to our policy

All data will be made available at https://osf.io/njd62/ soon after publication. We originally intended to make it available on 1 September, 2022, but we will treat this as a latest-posting date, and make clear in the data availability that we will share it with researchers that request it beforehand. The additional data used from secondary sources are: 1. Country classifications: https://datahelpdesk.worldbank.org/knowledgebase/articles/906519-world-bank-country-and-lending-groups.

2. Gross domestic product (in current US$). Data obtained from World Bank database (https://data.worldbank.org/indicator/NY.GDP.MKTP.CD)
3. GINI index. World Bank estimate. We used the latest data available retrieved from https://data.worldbank.org/indicator/SI.POV.GINI
4. Inflation: We used inflation as relative in consumer prices index (change in annual percentage) from the World Bank database (retrieved from https://data.worldbank.org/indicator/FP.CPI.TOTL.ZG) 5. The stimulus data used in Figure 1 is not publicly released as it belongs to a financial institution. Inquiries about accessing this data may be sent to arf25@columbia.edu.

Field-specific reporting

Please select the one below that is the best fit for your research. If you are not sure, read the appropriate sections before making your selection.

☐ Life sciences ☒ Behavioural & social sciences ☐ Ecological, evolutionary & environmental sciences

For a reference copy of the document with all sections, see nature.com/documents/nr-reporting-summary-flat.pdf

Behavioural & social sciences study design

All studies must disclose on these points even when the disclosure is negative.

Study description

A 61-country decision-study testing temporal discounting with an emphasis on economic inequality. All participants completed a survey of approximately 25-30 items, which was identical for all participants with a small number of contingent items.

Research sample

Entirely random sample of adults (locally-defined; typically 18 and older) from 61 countries (47% female; mean age = 34). Samples were not weighted or recruited in a way that ensured representativeness, but instead used the most random approach possible given the pandemic (i.e., all testing done online, typically on personal computers or at community centers in regions with low computer access). As explained in the next box, we targeted a sample of adults that would produce a sufficiently powered estimate for comparisons within and between countries. We only focused on adults due to the nature of the financial topics.

Sampling strategy

We use what we refer to as the Demic-Veckalov (named for Emir Demic and Bojana Veckalov) method for sampling: All collaborators used a range of circulation points, including email lists, discussion boards, and social media pages to recruit as random a sample as possible. This meant we primarily did not use individual pages to recruit, but instead, found recent posts with high engagement (often related to financial news) as well as common-interest platforms (e.g., Reddit channels). We also contacted NGOs and other organizations to assist with circulation. As described in the preregistration (https://osf.io/jfvh4), we identified a minimum sample size of 30 to achieve sufficient power (.95) for extremely small effects, though we aimed for over 120 participants as a minimum target for each country. The minimum of 30 was easily achieved for all countries included in the final version; a small number of countries did not meet the ideal 120. We also used a sliding scale target of 240 for countries of over 10 million population, and 360 for those over 100 million.

Data collection

All participants completed the study via Qualtrics; no researcher was present at the time of data collection and there were no conditions for blinding. Participants could choose the local national language or English (in some cases, additional languages were offered). No hard-copy versions were used. In Nepal and Ethiopia, we were informed that a community center may have hosted participants to support data collection, but this was organized outside the research team.

Timing

All surveys were collected between late July and early September 2021.

Data exclusions

We removed 6,141 participants (23.7%) who did not pass the attention check. 69 participants were removed for giving nonsensical responses to open data text (i.e., “helicopter” as gender). We removed 13 participants claiming to be over 100 years old. Based on the length of time for responses, 5,870 individuals that completed faster than three times the absolute deviations below the median time or that took less than 120 seconds to respond were removed. We further removed responses from IP addresses identified as either “tests” or “spam” by the Qualtrics service (264).

Non-participation

9,434 individuals did not complete at least 90% of the survey and were therefore excluded.

Randomization

No randomization was used apart from a small number of specific questions in the survey. All participants completed essentially the same version of the instrument.

Reporting for specific materials, systems and methods

We require information from authors about some types of materials, experimental systems and methods used in many studies. Here, indicate whether each material, system or method listed is relevant to your study. If you are not sure if a list item applies to your research, read the appropriate section before selecting a response.

Materials & experimental systems

n/a	Involved in the study
☒	☐ Antibodies
☒	☐ Eukaryotic cell lines
☒	☐ Palaeontology and archaeology
☒	☐ Animals and other organisms
☐	☒ Human research participants
☒	☐ Clinical data
☒	☐ Dual use research of concern

Methods

n/a	Involved in the study
☒	☐ ChIP-seq
☒	☐ Flow cytometry
☒	☐ MRI-based neuroimaging

Human research participants

Policy information about studies involving human research participants

Population characteristics	Participants were 47% female with a mean age of 34. Almost 100% of participants had completed some formal education, with 72% completing some form of higher education. 16% were current students. Over half (53%) had full-time employment; 10% were unemployed and 3% were retired. (See above for more.)
Recruitment	We use what we refer to as the Demic-Veckalov (named for Emir Demic and Bojana Veckalov) method for sampling: All collaborators used a range of circulation points, including email lists, discussion boards, and social media pages to recruit as random a sample as possible. This meant we primarily did not use individual pages to recruit, but instead, found recent posts with high engagement (often related to financial news) as well as common-interest platforms (e.g., Reddit channels). We also contacted NGOs and other organizations to assist with circulation. The primary forms of bias that this could create would be over-representation of individuals with computers/social media accounts, younger and more educated participants (due to the types of news stories often used as a conduit for recruiting), and individuals that speak the primary local language.
Ethics oversight	The study was approved by the Institutional Review Board at Columbia University in the City of New York.

Note that full information on the approval of the study protocol must also be provided in the manuscript.
